# Rational Design, Synthesis, Characterization and Evaluation of Iodinated 4,4′-Bipyridines as New Transthyretin Fibrillogenesis Inhibitors

**DOI:** 10.3390/molecules25092213

**Published:** 2020-05-08

**Authors:** Alessandro Dessì, Paola Peluso, Roberto Dallocchio, Robin Weiss, Giuseppina Andreotti, Mariateresa Allocca, Emmanuel Aubert, Patrick Pale, Victor Mamane, Sergio Cossu

**Affiliations:** 1 Institute of Biomolecular Chemistry ICB, CNR, Secondary Branch of Sassari, Traversa La Crucca 3, Regione Baldinca, Li Punti, 07100 Sassari, Italy; alessandro.dessi@cnr.it (A.D.); roberto.dallocchio@cnr.it (R.D.); 2 Strasbourg Institute of Chemistry, UMR CNRS 7177, Team LASYROC, 1 rue Blaise Pascal, University of Strasbourg, CEDEX, 67008 Strasbourg, France; robin.weiss@unistra.fr (R.W.); ppale@unistra.fr (P.P.); 3 Institute of Biomolecular Chemistry ICB, CNR, Via Campi Flegrei 34, 80078 Pozzuoli (NA), Italy; giuseppina.andreotti@icb.cnr.it (G.A.); mariateresa.allocca@gmail.com (M.A.); 4 Department of Environmental, Biological and Pharmaceutical Sciences and Technologies (DiSTABiF), University of Campania “Luigi Vanvitelli”, Via Vivaldi, 43-81100 Caserta, Italy; 5 Crystallography, Magnetic Resonance and Modelling (CRM2), UMR CNRS 7036, University of Lorraine, Bd des Aiguillettes, 54506 Vandoeuvre-les-Nancy, France; emmanuel.aubert@univ-lorraine.fr; 6 Department of Molecular Science and Nanosystems DSMN, Venice Ca’ Foscari University, Via Torino 155, 30172 Mestre Venezia, Italy; cossu@unive.it

**Keywords:** bipyridines, docking, fibril formation, halogen bond, misfolding inhibition, transthyretin

## Abstract

The 3,3′,5,5′-tetrachloro-2-iodo-4,4′-bipyridine structure is proposed as a novel chemical scaffold for the design of new transthyretin (TTR) fibrillogenesis inhibitors. In the frame of a proof-of-principle exploration, four chiral 3,3′,5,5′-tetrachloro-2-iodo-2′-substituted-4,4′- bipyridines were rationally designed and prepared from a simple trihalopyridine in three steps, including a Cu-catalysed Finkelstein reaction to introduce iodine atoms on the heteroaromatic scaffold, and a Pd-catalysed coupling reaction to install the 2′-substituent. The corresponding racemates, along with other five chiral 4,4′-bipyridines containing halogens as substituents, were enantioseparated by high-performance liquid chromatography in order to obtain pure enantiomer pairs. All stereoisomers were tested against the amyloid fibril formation (FF) of wild type (WT)-TTR and two mutant variants, V30M and Y78F, in acid mediated aggregation experiments. Among the 4,4′-bipyridine derivatives, interesting inhibition activity was obtained for both enantiomers of the 3,3′,5,5′-tetrachloro-2′-(4-hydroxyphenyl)-2-iodo-4,4′-bipyridine. In silico docking studies were carried out in order to explore possible binding modes of the 4,4′-bipyridine derivatives into the TTR. The gained results point out the importance of the right combination of H-bond sites and the presence of iodine as halogen-bond donor. Both experimental and theoretical evidences pave the way for the utilization of the iodinated 4,4′-bipyridine core as template to design new promising inhibitors of TTR amyloidogenesis.

## 1. Introduction

**Figure 1:**
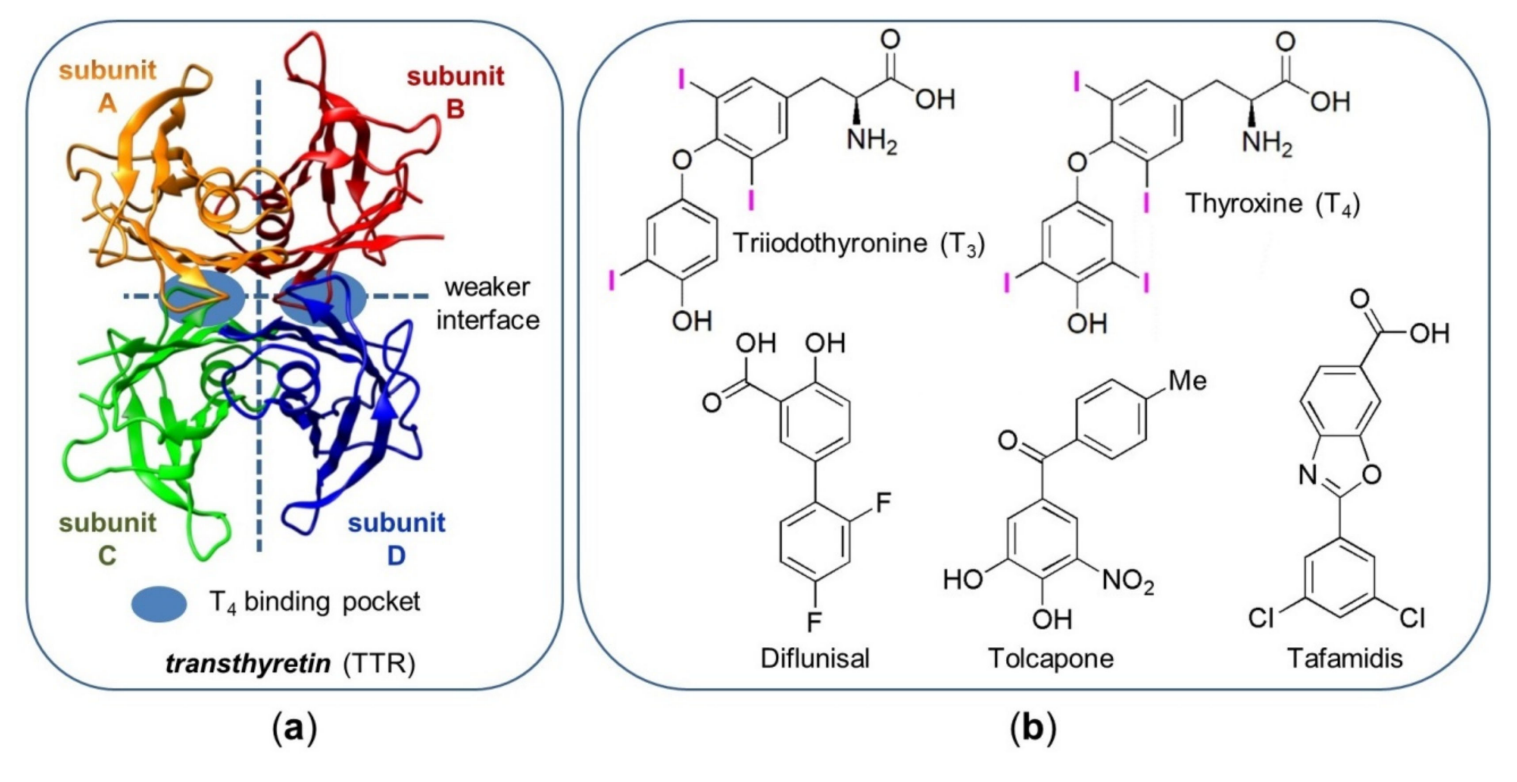
(**a**) Ribbon diagram picture of wild type (WT)-TTR (released from PDB ID 1ICT [2]); (**b**) structures of thyroxine (T_4_), triiodothyronine (T_3_), tafamidis and other ATTR drug candidates under clinical trials.

Human transthyretin (hTTR, prealbumin) is a homotetrameric protein (55 kDa) made up of four subunits (A-B-C-D, Figure 1a), each composed of 127 aminoacids [1,2,3]. The protein is mainly produced by the liver, but other sources of TTR, including the choroid plexus, the retinal pigment epithelium, and the α-cells of pancreatic islets are known. TTR is involved in the transport of thyroid hormones (thyroxine (T_4_) and triiodothyronine (T_3_)) (Figure 1b) in blood and cerebrospinal fluid. The tetrameric assembly of TTR contains a central hydrophobic channel with two symmetrical funnel-shaped T_4_ binding pockets located at the energetically weaker dimer-dimer A-B/C-D interface [4], the other A-C/B-D interface being stabilized by interstrand hydrogen bonds (HBs).

The presence of mutations in TTR results in destabilization of the tetramer and dissociation into amyloidogenic monomers, which misfold and self-aggregate into insoluble amyloid fibrils. TTR amyloidogenesis has been implicated to cause amyloid diseases (TTR amyloidosis, ATTR) [5,6]. Amyloid structures derived from wild-type (WT)-TTR can deposit in the heart and peripheral nerves causing senile systemic amyloidosis (SSA), whereas over 100 TTR mutants were shown to be implicated in three other groups of diseases, namely familial amyloidotic polyneuropathy (FAP), cardiomyopathy (FAC), and central nervous system selective amyloidosis (CNSA). Among known TTR mutants, the V30M [7] and the Y78F [8] are the most common FAP mutation and the most amyloidogenic, respectively. Moreover, the importance of TTR stability also concerns neurodegenerative disorders such as Parkinson’s and Alzheimer’s diseases [9,10]. 

In 1996 it was observed that T_4_ is able to prevent amyloid fibril formation (FF), but with adverse side effects due to its hormonal activity [11]. However, this study proved that stabilization of the native TTR tetramer by small molecule binding to one or both of the T_4_ binding pockets could be a rationale for the kinetic inhibition of TTR amyloidogenesis, tetramer dissociation being the rate-determining step in amyloid formation [12,13]. Indeed, TTR can bind molecules other than T_4_ because more than 99% of T_4_ binding sites are unoccupied due to the fact that two other carriers, albumin and thyroid-binding globulin, transport the majority of T_4_ in the blood. Significantly, this observation paved the way for the development of an oral therapy as a valid alternative to more invasive approaches [14].

In the last decades, several small molecules have proven to be able to bridge neighbouring monomers of TTR via specific hydrophobic and electrostatic noncovalent interactions, to stabilize the tetramer, to inhibit the tetramer dissociation, and to reduce FF [3,15,16,17,18,19,20,21,22,23,24,25,26,27,28]. However, to date few structures possess the requirements to be drug candidates (Figure 1b), and the ATTR still lacks an effective therapy. So far, tafamidis [29] has been approved for clinical use in Europe and Japan for the treatment of early-stage FAP [30], but advanced disease responds poorly to tafamidis administration [31]. Then, recently the drug tolcapone, which was approved for the treatment of Parkinson’s disease, has been repurposed for the treatment of ATTR [32]. Nonsteroidal anti-inflammatory drugs (NSAIDs) also inhibit formation of amyloidogenic monomers. Among them, diflunisal was shown to slow the progression of FAP over a period of 2 years, but adverse gastrointestinal effects limit its use [16,33]. In this regard, it is worth mentioning that small molecules should be selective for TTR and neither bind thyroid hormone receptors nor cyclooxygenase-1 (COX-1) to be effective clinical candidates [16,18].

Over time, a large number of SAR studies have been published in order to identify typical substructures which are shared by compounds that stabilize TTR tetramer [3,34,35,36,37,38,39]. In this field, the potential of iodine as substituent (iodination hypothesis) has been intensively explored through evaluating the inhibition ability of iodinated compounds with respect to non-iodinated analogues [10,11,17,36,40,41,42,43]. Among iodinated motifs, 2,4,6-triiodophenol proved to be one of the most potent TTR fibrillogenesis inhibitor known so far [11], and iododiflunisal was shown to stabilize TTR [17]. Nevertheless, in some cases iodination does not improve inhibition activity [17,42,44], the effectiveness of this strategy depending on inhibitor scaffold structure and location of iodine in the molecular framework [42]. In general, the real function of iodine in TTR binding and stabilization still remains unclear due to the fact that halogens as substituents can contribute to protein-ligand binding through multiple roles such as hydrophobic centres, HB acceptors and electrophilic halogen bond (XB) donors. In particular, XBs were found to have a relevant role in the binding of halogenated ligands with proteins [42,45], and short I···O contacts were found to underlie the selective binding of T_4_ to TTR [46]. Despite the potential of iodine substituents to contribute to TTR stabilization, studies on iodinated compounds are reported in smaller degree compared to compounds containing fluorine, chlorine and bromine as substituents. Currently, the scarce availability of iodinated compounds has clearly limited the study of the iodine effect on TTR misfolding inhibition, reducing the possibility to gather conclusive information on iodine function. 

**Figure 2:**
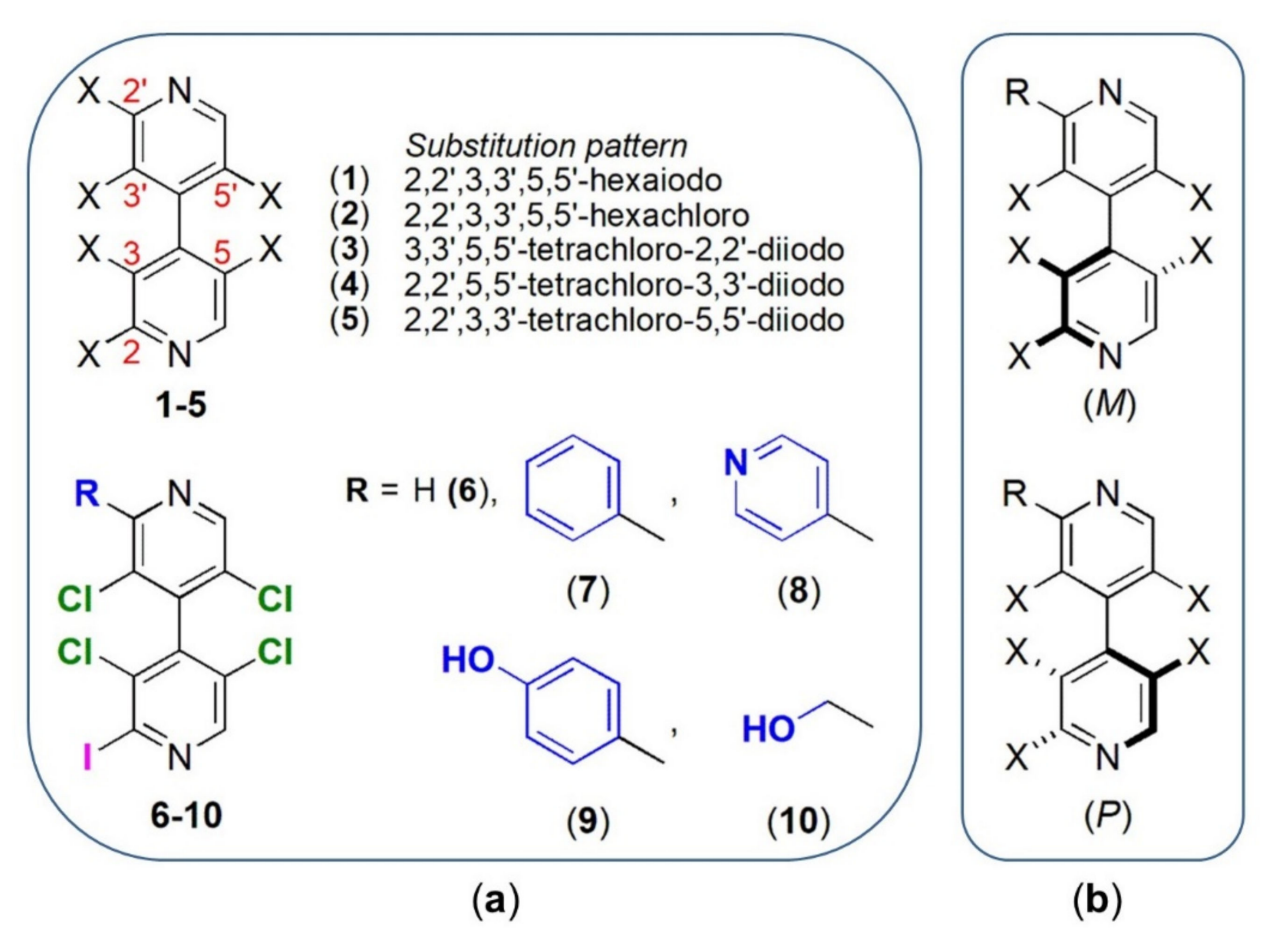
(**a**) Structures of halogenated 4,4′-bipyridines **1**–**10**; (**b**) (*M*) and (*P*) absolute configurations of the atropisomeric 4,4′-bipyridines **1**–**5** and **7**–**10**.

In the last few years, our groups have reported the synthesis and characterization of halogenated 4,4′-bipyridines [47,48,49,50]. These molecules were shown to be efficient as XB donors in the solid state [48] and in solution [51,52]. On this basis, starting from the chemistry and properties of 4,4′-bipyridines **1**–**5** [48], we describe herein the design and synthesis of iodinated 4,4′-bipyridines **6**–**10** and their evaluation as TTR fibrillogenesis inhibitor (Figure 2a). Due to the atropisomeric geometry of their halogenated heteroaromatic scaffold, compounds **1**–**5** and **7**–**10** are chiral and exhibit (*M*)- and (*P*)-enantiomers (Figure 2b). Quantitative evaluation of the inhibition efficacy of both (*M*)- and (*P*)-enantiomers against TTR misfolding was performed through acid-mediated in vitro assays of WT-, V30M-, and Y78F-TTR aggregation. The relationship between chemical structures, inhibition activity, and binding mode was explored by molecular docking and electrostatic potential isosurfaces (*V*_S_) analysis.

In particular, this study aims at gathering information on the potential role of substructures containing halogen σ-holes in TTR binding and stabilization. It is noteworthy that halogenated 4,4′-bipyridines derivatives have never been designed nor tested as TTR fibrillogenesis inhibitors.

## 2. Results and Discussion

### 2.1. Rational Design

**Figure 3:**
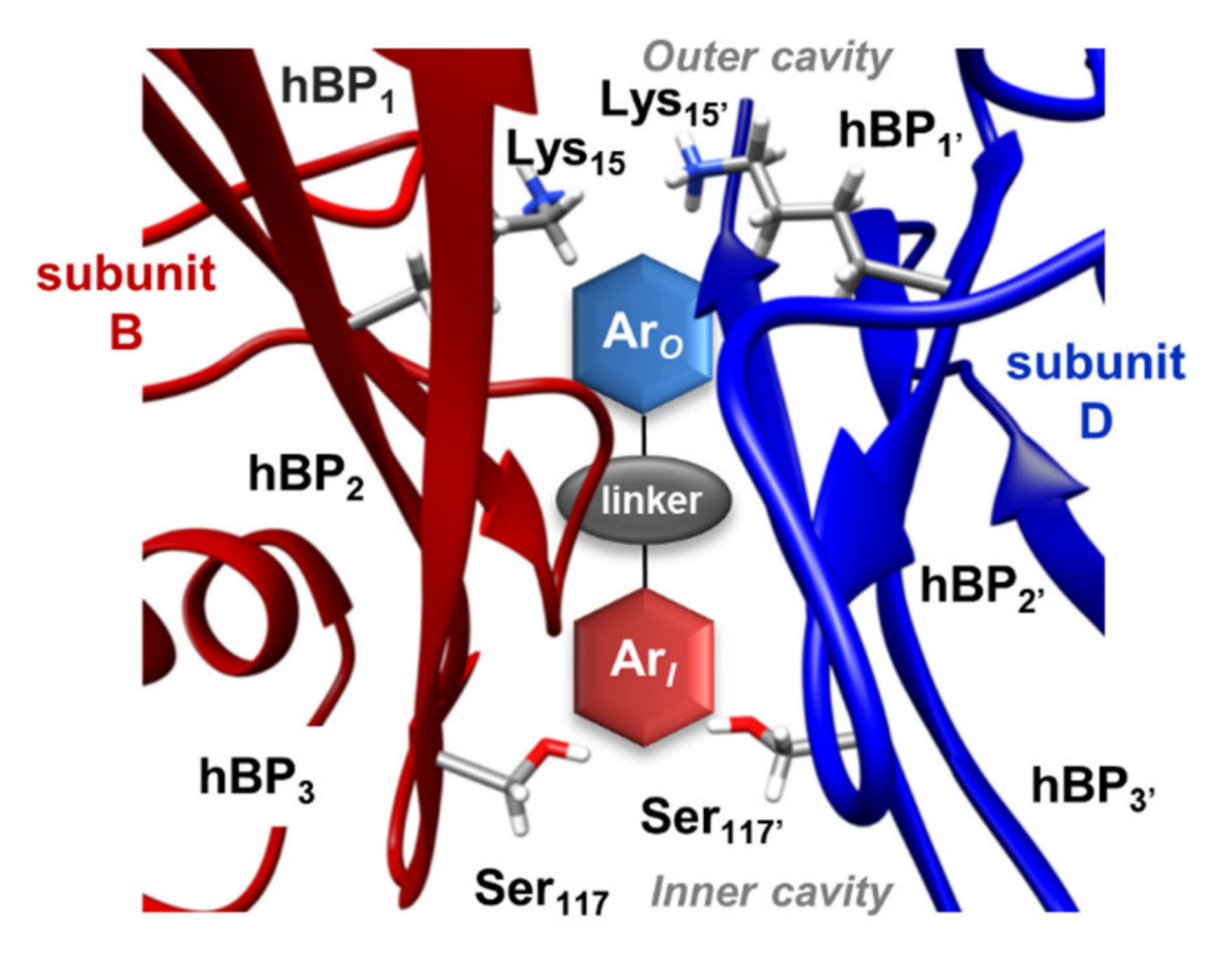
Expanded schematic view of one of the two T_4_ binding pockets (dimer BD), where hBP_1_ is located between the side chains of Lys_15_/_15’_, Leu_17_/_17’_, Thr_106_/_106’_ and Val_121_/_121’_, hBP_2_ between the side chains of Leu_17_/_17’_, Ala_108_/_108’_, Ala_109_/_109’_ and Leu_110_/_110’_ and hBP_3_ between the side chains of Ser_117_/_117’_, Thr_119_/_119’_, Ala_108_/_108’_ and Leu_110_/_110’_.

In the majority of the TTR:small molecule crystal structures reported to date [3], the molecule is bound to the T_4_ binding site, with the exception of polyphenol (−)-epigallocatechin gallate (EGCG) which binds TTR molecular surface [19] and few other allosteric inhibitors [24,25]. Each T_4_ binding pocket is composed of a small inner cavity (delimited by the pair Ser_117_/Se_117’_), and a large outer cavity (delimited by the pair Lys_15_/Lys_15’_), with three pairs of symmetric hydrophobic depressions named halogen binding pockets (hBPs) (Figure 3) where the iodine atoms of T_4_ are located.

As reported by Kelly and co-workers [53], typical inhibitors are characterized by two aryl rings (Ar*_O_* and Ar*_I_*) connected directly or through a linker. The binding ability of this kind of molecules can be modulated by tuning structure and substitution pattern of the three substructures, namely Ar*_O_*, Ar*_I_* and linker. Scaffolds bearing halogen substituents on one ring, and a HB centre on the other ring, which is able to interact with Lys_15_ ε-NH_3_^+^ group, represent the typical motif of most inhibitors reported so far. In general, TTR can accommodate small molecules with different orientations. Indeed, in the forward binding mode, the phenyl ring substituted with halogens prefers the inner cavity, whereas in the reverse binding mode it is located in the outer cavity [3,15,16].

#### 2.1.1. Conceptual Basis

**Figure 4:**
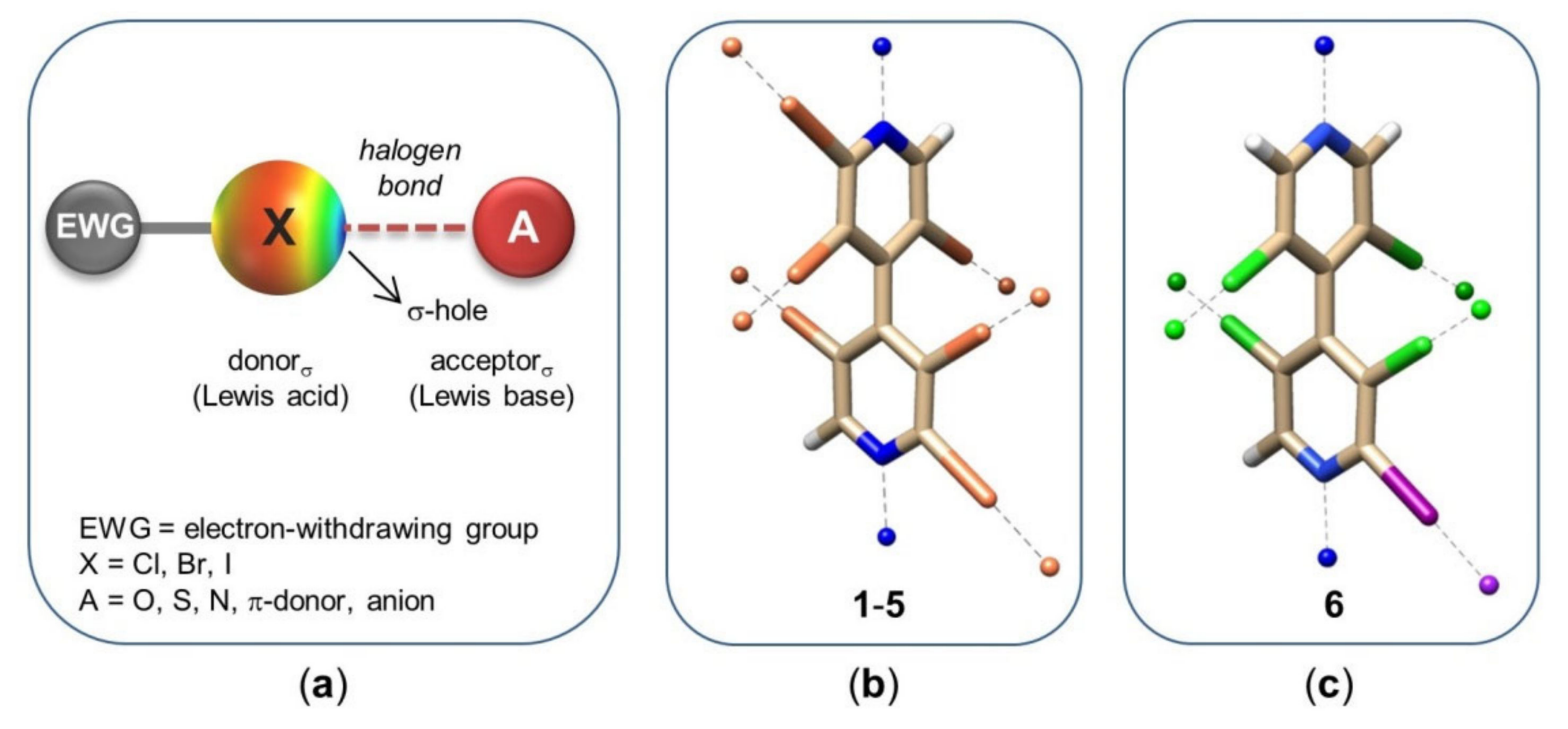
(**a**) Schematic description of XB. On X surface, electrostatic potential (*V*_S_) > 0 (lower electron density) and *V*_S_ < 0 (higher electron density) are indicated in blue and red, respectively. Intermediate values are in orange, yellow, and green; (**b**) and (**c**) positions of nitrogen *V*_S,min_ regions (blue) and *V*_S,max_ in the structure of halogenated 4,4′-bipyridines **1**–**5** and **6** (hydrogen, white). *V*_S,max_ colors: halogen, coral; chlorine, green; iodine, purple.

As reported [3,42,46], the possibility of XB formation emerges from the crystallographic analysis of complexes between TTR and some of the halogenated ligands reported so far. Indeed, contacts ranging from 2.8 to 3.5 Å have been observed between halogen substituents of small molecules and Ala_109_, Ser_117_, and Thr_119_ carbonyls in TTR, acting as XB acceptors. The XB is a noncovalent interaction which originates from the anisotropic charge distribution of bound halogens, generating an area of lower electron density, the electrophilic σ-hole, located on the elongation of the covalent bond (Figure 4a) [54].

From this perspective, the electrostatic potential (*V*) is a useful tool for understanding molecular interactive capability. In particular, the calculated positive *V* on the isodensity surface near the halogen σ-hole, the *V*_S,max_, is advantageously used to assess σ-hole depth which, in turn, has been found to be related to the strengths of XB [55,56]. On this basis, the *V*_S_ values on a 0.002 au molecular isosurface of polyhalogenated 4,4′-bipyridines **1**–**6**, bearing iodines and chlorines as substituents, were calculated in order to localize regions of higher (negative *V*_S,min_) and lower (positive *V*_S,max_) electron charge density, and to profile the binding capability of diverse halogenated 4,4′-bipyridyl scaffolds as templates for the design of new TTR stabilizers (Figure 4b,c). Several key structural aspects in halogenated 4,4′-bipyridines highlight the interest of studying this family of molecules:

(i) 4,4′-Bipyridines **1**–**6** consist of two linked heteroaromatic rings that accommodate six (**1**–**5**) and five (**6**) (Figure 4b,c) highly oriented halogens as substituents. 4,4′-Bipyridyl derivatives have been almost unexplored in the field of TTR stabilization to date, and only the 5-cyano-2-methyl-3,4’-bipyridin-6(1*H*)-one milrinone was found to be a strong competitive inhibitor of T_4_ binding to TTR [57];
(ii) The two electron-poor bipyridyl rings are characterized by positive *V*_S,max_ ranging from 72.06 to 96.58 kJ/mol (Supplementary Materials, Table S1), similar to the values calculated for tafamidis, which contains an electron-poor substructure centred on the oxazole ring (98.49; 98.88 kJ/mol) (Table S2);
(iii) The presence of four 3,3′,5,5′-substituents induces chirality by restricted rotation around the 4,4′-bond (atropisomerism), introducing stereochemical features that could be fruitfully exploited to improve binding efficacy and selectivity toward TTR. So far, few studies considered chiral inhibitors and their stereochemical properties [35]. Moreover, the atropisomeric structure of the 3,3′,5,5′-tetrasubstituted-4,4′-bipyridyl ring mimics the skewed conformation of T_4_;
(iv) The two pyridyl nitrogens can work as HB acceptors, exhibiting *V*_S,min_ values ranging from −122.43 to −117.07 kJ/mol (Supplementary Materials, Table S1). Furthermore, nitrogen is known to be a high-impact design element because substitution of a CH group with a N atom, in aromatic and heteroaromatic ring frameworks, often improves the pharmacological profile of a molecular structure at different levels [58];
(v) Iodine is considered a powerful XB donor due to its polarizability, in particular when it is bound to electron-withdrawing groups (EWGs). T_4_ presents multiple iodine atoms which are accommodated in the hBPs of the T_4_ binding site of TTR. For T_4_, we calculated *V*_S,max_ ranging from 109.11 to 143.05 kJ/mol (Supplementary Materials, Table S2). In compounds **1** and **3**–**6**, iodine substituents are activated as XB donors due to the EWG effect exerted by the tetrachlorinated heteroaromatic scaffold, with *V*_S,max_ values ranging from 133.42 to 167.06 kJ/mol (Table S1). In this regard, it is worth mentioning that in our previous studies [48,49,50,51,52,59], the function of compounds **1**–**5** as XB donors was demonstrated by theoretical calculations, and HPLC, NMR, and X-ray diffraction (XRD) analysis involving oxygen and nitrogen as XB acceptors. On the other hand, iodine is able to fill the hBPs better than other halogens due to its volume, so optimizing the strength of van der Waals interactions [42];
(vi) In compounds **2**–**6**, the chlorine substituents could also function as potential XB donors, even if lower *V*_S,max_ values ranging from 70.96 to 98.81 kJ/mol were calculated (Supplementary Materials, Table S1) due to the lower polarizability and the higher electronegativity of chlorine with respect to iodine. Moreover, COX-1 being characterized by smaller hydrophobic binding pockets with respect to TTR [34,35], the presence of bulkier chlorines, with respect to hydrogens, might reduce binding affinity of the small molecule towards COX-1, thus increasing selectivity in TTR binding;
(vii) Since compounds **3**–**5** contain the same type and number of halogenated substituents but a different substitution pattern, it is possible to evaluate the scaffold effect on iodine properties and activity. Recently, Boeckler and co-workers demonstrated by theoretical calculations that the attachment position of a halogen within a heteroaromatic scaffold can influence size and shape of the σ-hole and, in turn, the strength of the XB [56];
(viii) All haogens can also act as hydrophobic sites and HB acceptors due to a belt of higher electron density located around the σ-hole (Figure 4a).

#### 2.1.2. Molecular Docking of Polyhalogenated 4,4′-Bipyridines **1**–**6** in the TTR Tetramer 

Molecular docking is one of the most popular computational approaches in structure-based drug design [60]. This technique can be used either to identify the correct conformation of the ligand within the target binding pocket or to estimate the interaction energy between a target and a ligand [61,62]. Molecular dynamics (MD) simulations can be also advantageously used to evaluate the molecular flexibility of ligands and receptors [63]. However, as some conformational changes occur in the time scale of only dozens of nanoseconds, this aspect could compromise the MD simulation viability for virtual screening studies. Moreover, computational cost of MD simulations is generally high. Therefore, a useful theoretical strategy is to select promising conformations from the docking study. In this regard, it is worth mentioning that different programs and theoretical methods are available for docking purposes, and each docking program shows advantages and limitations to carry out docking studies and docking-based virtual screening [64,65,66,67]. On the other hand, Hou and co-worker recently illustrated that no single docking program has dominative advantages than other programs [60,67]. Herein, due to the novelty of the 4,4′-bipyridyl motif in this field, a blind docking [21] exploration was performed in order to inspect the disposition of all enantiomers (*M*) and (*P*) of compounds **1**–**5** and the achiral **6** into the whole TTR, focusing on the binding capability toward the T_4_ pockets. Despite the fact that flexible docking is frequently used to model ligand-receptor complexes accounting for molecular flexibility [64,65], we do not consider protein flexibility herein, rather focusing on the capability of the new compounds to insert in the same T_4_ binding cavity hosting T_4_, as derived from the selected crystal complex. For this purpose, the released crystal structure of TTR complex (PDB ID: 1ICT, chains ABCD) [2] from the RCSB protein databank (www.rcbs.org) was used. AutoDock 4.2.6 was employed as docking program [68,69], and the software Chimera 1.13.1 [70] for the graphical representation of the poses derived from the docking calculation. Moreover, due to the atropisomeric features of the selected compounds, ligand flexibility is rather limited in this case. With the aim to assess the electrophilic behaviour of the halogens, the extra point [71,72] or explicit σ-hole (ESH) (Supplementary Materials, Table S7) [73,74] concept was used to model the XB [51] in TTR/4,4′-bipyridine complexes. In the context of drug design, the use of the ESH correction to model XB in drug-receptor or drug-enzyme interactions has been recently reported [75]. 

**Figure 5:**
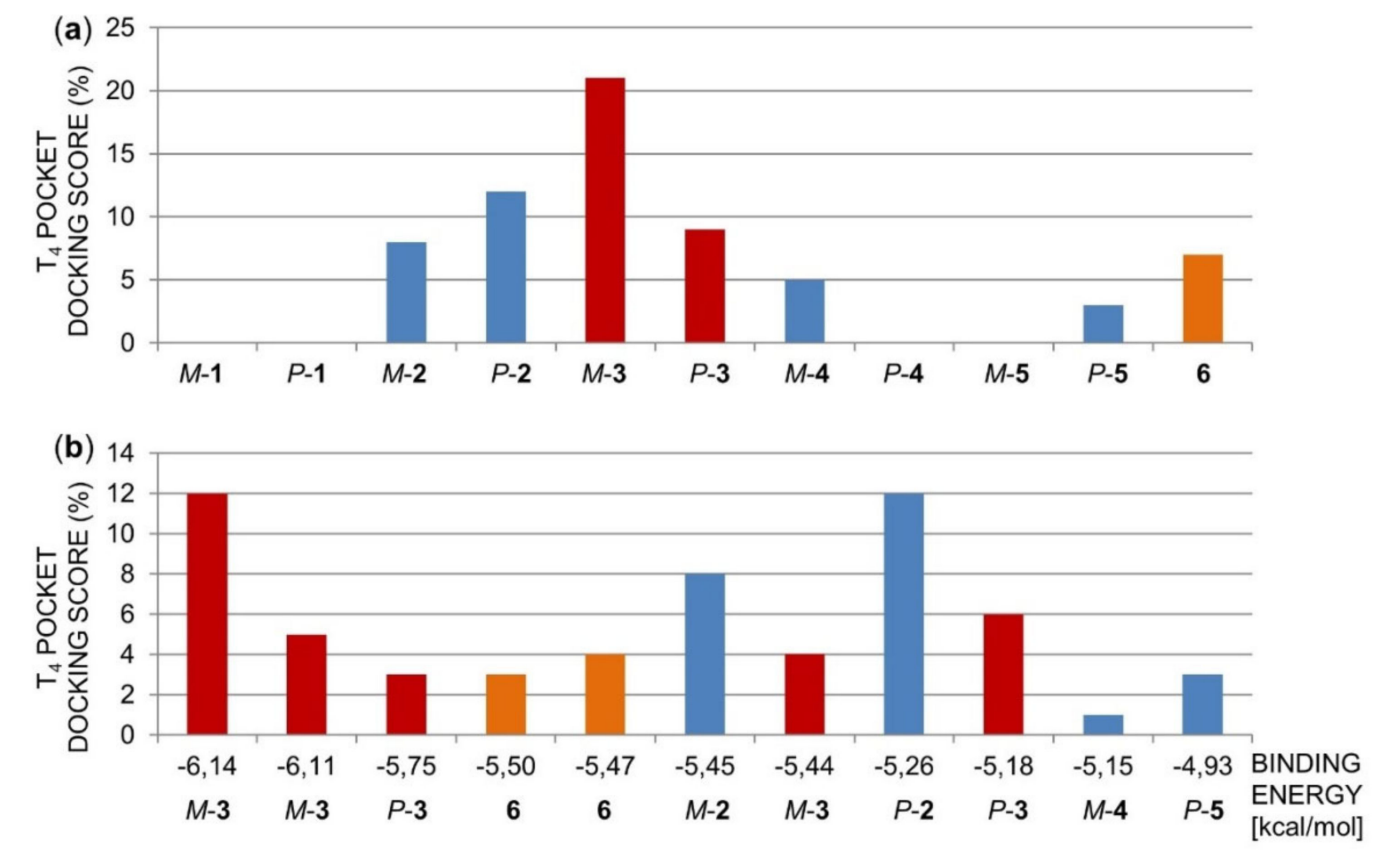
(**a**) Blind docking scores for the enantiomers of compounds **1**–**5** and the achiral **6** in the T_4_ pockets; (**b**) docking score and binding energies of each pose of **1**–**6** laying in the T_4_ pockets.

For both enantiomers of **1**, (*P*)-**4** and (*M*)-**5**, all poses laid outside the T_4_ binding pockets, and on the surface of only one monomer. 4,4′-Bipyridines **2**, **3**, **6** and the enantiomers (*M*)-**4** and (*P*)-**5** were found in the T_4_ pockets with docking score percentages lower than 21% and binding energy ranging from −6.14 to −4.93 kcal/mol. In particular, among all sites emerged from the blind docking procedure, the T_4_ pockets were found to be the lowest energy sites for all compounds (Supplementary Materials, Table S8). By comparing the clustering of the docking poses of the (*M*) series, it could be observed a clear preference of **3** for the T_4_ pockets compared to compounds **4** and **5** (Figure 5a). Indeed, 21% of docking poses of (*M*)-**3** laid inside the T_4_ binding pockets, whereas lower percentages resulted for **2** and **4** (8% and 5%, respectively), these poses anchoring the side chains of the amino acid residues which identify the three hBPs (Supplementary Materials, Table S8 and Figure S2). The lowest binding energy values (Figure 5b) were observed for all poses of compounds **3** and **6** in the T_4_ pockets, thus the 3,3′,5,5′-tetrachloro-2-iodo-4,4′-bipyridine motif showed higher binding capability for the T_4_ pocket with respect to the other halogenated scaffolds. With the aim to confirm the docking results, all compounds **1**–**6** were tested in a first series of TTR FF assays.

#### 2.1.3. Acid-Mediated TTR FF Assay for Compounds **1**–**6** and Structure-Activity Relationships

Simulating the conditions found in the lysosome, the treatment of transparent solutions of tetrameric TTR with acids induces FF and precipitation that can be observed in vitro [15]. As FF is proportional to the turbidity degree of the parent solution, the inhibition capability of small molecules tested as fibrillogenesis inhibitors can be derived from turbidimetry analysis [76]. On this basis, the enantiomers of compounds **1**–**5** and the 2-iodinated-4,4′-bipyridine **6** were tested in this acid-mediated TTR fibrillogenesis assay in order to evaluate their capability to inhibit FF and precipitation. Both WT- and the mutant Y78F-TTR [8,77] were used as assay proteins. Thus, the compounds under evaluation were first incubated for 30 min with TTR at neutral pH (36 °C). The pH was then lowered to give the maximal rate of FF for a given variant (4.4 for WT, 5.0 for Y78F), and the samples were incubated for 72 h at 36 °C. For each sample, the degree of FF was determined by comparing its turbidity at 400 nm to that of a sample of TTR without test compound. On this basis, FF percentage (FF%) characterizes compound activity. Thus, the capability of compounds **1**–**6** in terms of FF% are summarized in Table 1. The assays were carried out at two inhibitor concentrations, namely 7.2 μM (TTR/inhibitor ratio 1:2) and 3.6 μM which is equal to the TTR concentration in plasma. diflunisal was used as the positive control, whereas TTR under acidic conditions, without inhibitor, was fixed as a negative control (100% FF mark). On this basis, 10% FF corresponds to a compound inhibiting 90% of TTR fibrillogenesis.

With WT-TTR, high degree of fibrillogenesis was observed for compounds **1**–**6** with FF% ranging from 90 to 100%, whereas with the mutant variant Y78F slightly lower values were found in almost all cases. In accord with the docking evaluations, in general the (*M*)-enantiomers were found to be slightly more effective than the (*P*)-enantiomers, whereas both enantiomers of compound **1** showed no inhibition activity against fibrillogenesis. Interestingly, compounds (*M*)-**3** and **6** proved to be the best inhibitors against Y78F-TTR fibrillogenesis with values ranging from 62 to 82%.

Compounds **1**–**6** were also docked in the T_4_ pocket at the BD interface in order to derive the respective estimated free energy of binding (EFEB) (Supplementary Materials, Table S9). The crystallographic T_4_ structure [2] was used as benchmark ligand to validate the docking procedure (Supplementary Materials, Figure S3). The predictive success of the EFEB values was verified by linear regression. 

**Figure 6:**
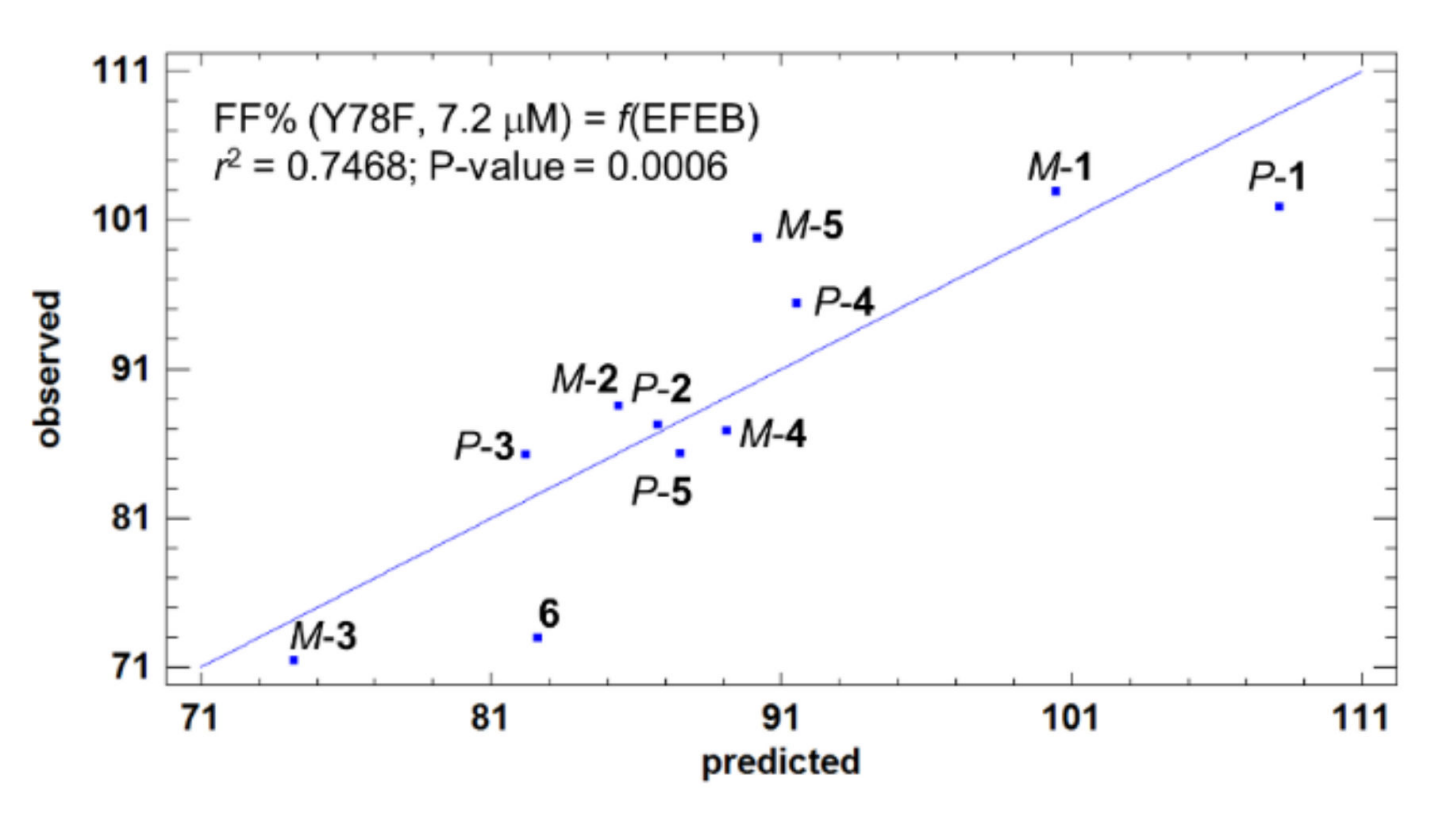
Linear regression analysis describing the relationships between FF% (Y78F-TTR, inhibitor concentration = 7.2 μM) and calculated EFEB by docking in the T_4_ pocket (BD).

For enantiomers **1**–**5** and compound **6**, the results of fitting a simple linear regression model to describe the relationship between FF% and EFEB, as an independent variable, is reported in Figure 6 for Y78F at 7.2 μM (Supplementary Materials, Figure S4 for the plots of WT-TTR and Y78F at 3.6 μM). In all cases, the *P*-values of the considered independent variable are lower than the statistical benchmark value of 0.05.

It is worth mentioning that no statistical correlation was found between FF% and the EFEB values calculated without the application of the ESH parametrization for halogens (0.1199 ≤ *P*-value ≤ 0.9871).

The complete inactivity of compound **1** could be related to the six bulky iodine substituents which prevent the accommodation of the molecule in the T_4_ pocket. From a comparative analysis of the structure-activity relationships, it seems that the presence of iodine substituents at positions 3,3′,5,5′ (compounds **1**, **4**, **5**) is detrimental for both binding and inhibition activity, whereas 2- and 2′-iodine substitutions exhibit a beneficial effect on 4,4′-bipyridine activity.

From these results, the 3,3′,5,5′-tetrachloro-2-iodo-4,4′-bipyridyl substructure (iodine *V*_S,max_ values: 140.8 kJ/mol for **3**, 136.8 kJ/mol for **6**) has been identified as the most interesting scaffold for elaborating more specific compounds. On this basis, compounds **7**–**10** were prepared by focused procedures to introduce a phenyl, a 4-pyridyl, a 4-hydroxyphenyl and a hydroxymethyl as a 2′-substituent, respectively. These substituents were selected to probe how subtle structural changes at position 2′ can influence the inhibition activity.

### 2.2. Chemistry

#### 2.2.1. Syntheses of 4,4′-Bipyridines **1**–**10**

The synthesis of chiral hexahalogenated 4,4′-bipyridines **1**–**5** was previously reported by our groups, exploiting convergent or divergent strategies depending on halogen type and position on the heteroaromatic scaffold [47,48]. These methodologies proved to be extremely versatile and allowed to introduce Cl, Br, and I at any position of the 4,4′-bipyridyl scaffold [48].

**Scheme 1:**
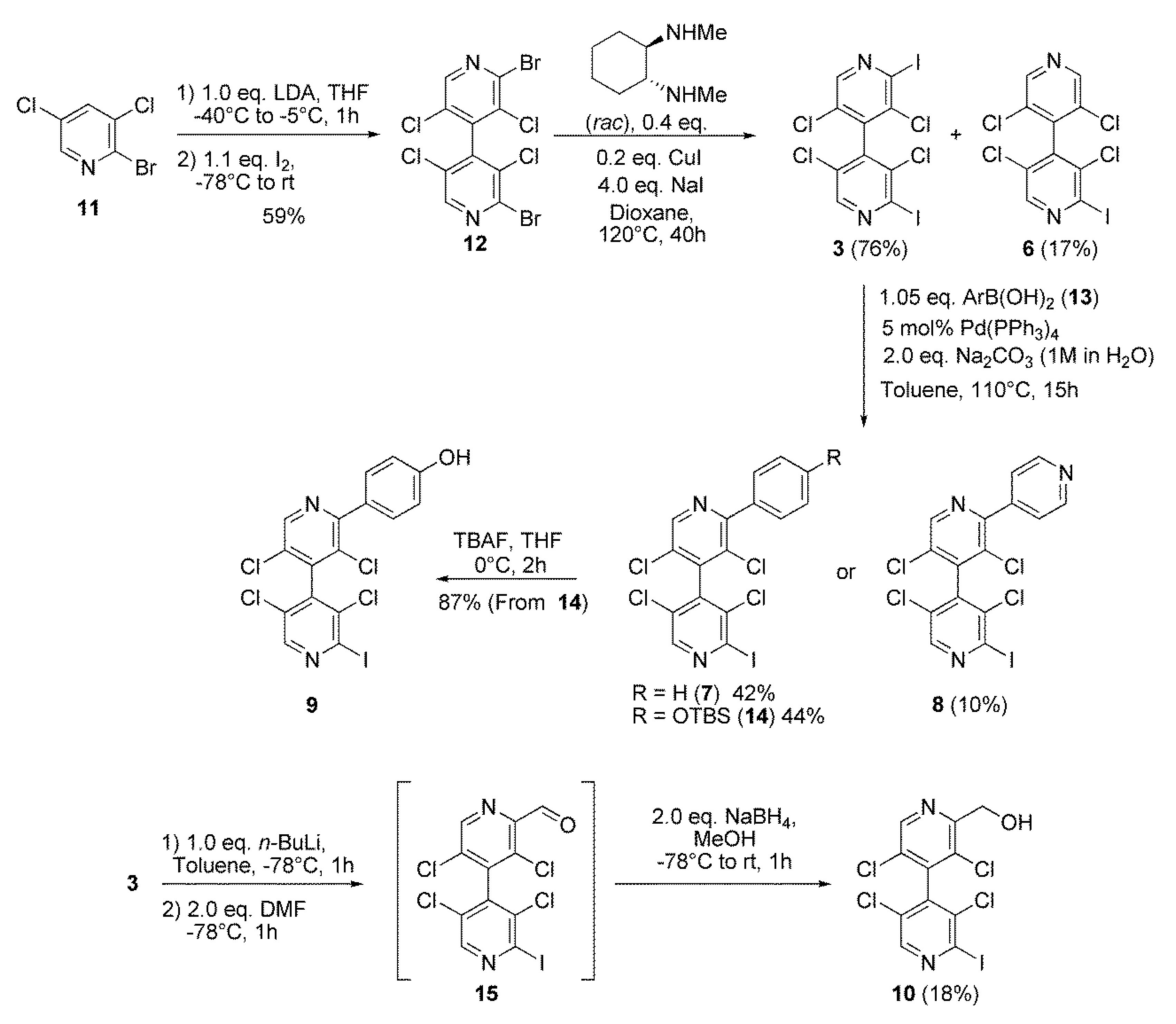
Synthesis of bipyridines **6**–**10**.

3,3′,5,5′-Tetrachloro-2,2′-diiodo-4,4′-bipyridine (**3**) was prepared in two steps from the commercially available 2-bromo-3,5-dichloropyridine (**11**). First, a lithium diisopropylamide (LDA)-mediated dimerization afforded 3,3′,5,5′-tetrachloro-2,2′-dibromo-4,4′-bipyridine (**12**), and then the bromine atoms in 2,2′-positions were exchanged for iodines through a diaminocopper-catalyzed Finkelstein reaction. Along with the expected 4,4′-bipyridine **3**, careful chromatographic purification allowed obtaining in small quantity the 3,3′,5,5′-tetrachloro-2-iodo- 4,4′-bipyridine **6** (Scheme 1).

For 4,4′-bipyridines **7**–**9**, the aromatic ring in 2-position was introduced through a Suzuki coupling reaction involving 4,4′-bipyridine **3** and one equivalent of the respective boronic acid **13**. The use of phenyl- and 4-pyridylboronic acids **13a,b** afforded 3,3′,5,5′-tetrachloro-2-iodo-2′-phenyl-4,4′-bipyridine (**7**) and 3,3′,5,5′-tetrachloro-2-iodo-2′-(4- pyridyl)-4,4′-bipyridine (**8**), respectively.

The direct synthesis of the 3,3′,5,5′-tetrachloro-2-iodo-2′-(4-hydroxyphenyl)-4,4′-bipyridine (**9**) by coupling of **3** with 4-hydroxyphenylboronic acid (**13c**) failed under Suzuki coupling conditions. Therefore, compound **9** was obtained in two steps by using the *tert*-butyldimethylsilyl (TBS)-protected 4-hydroxyphenylboronic acid **13d**. After the cross-coupling reaction between compounds **3** and **13d**, 3,3′,5,5′-tetrachloro-2-iodo-2′-(4-*tert*-butyldimethylsilyloxyphenyl)- 4,4′-bipyridine (**14**) was isolated, and the TBS-protecting group was subsequently removed by using a tetrahydrofuran solution of tetrabutylammonium fluoride (*n*-Bu_4_NF), affording 4,4′-bipyridine **9**. Finally, 3,3′,5,5′-tetrachloro-2-iodo-2′-hydroxymethyl-4,4′-bipyridine (**10**) was obtained in a one-pot two-step process starting from 4,4′-bipyridine **3**. After iodine-lithium exchange with one equivalent of *n*-butyllithium (*n*-BuLi) in toluene, the lithiated species was quenched with dimethylformamide (DMF) to give the aldehyde intermediate **15** which was directly reduced with sodium borohydride (NaBH_4_) to afford bipyridine **10**.

#### 2.2.2. Enantioseparation of 4,4′-Bipyridines **7–10** on Chiral Stationary Phases

*Rac*-4,4′-bipyridines **1**–**5** were enantioseparated at multimilligram scale on polysaccharide- based chiral stationary phases, as previously reported by our groups [48,59]. Pure enantiomers (>95% ee) of 3,3′,5,5′-tetrachloro-2-iodo-2′-substituted-4,4′-bipyridines **7**–**10** were obtained in good amounts by means of HPLC multimilligram enantioseparation under normal phase elution conditions (Supplementary Materials, Table S10). In particular, the enantiomers of compounds **7** and **9** were recovered by using Chiralpak IA (immobilized amylose tris(3,5-dimethyl- phenylcarbamate)) as a chiral column, whereas the Chiralcel OD-H (cellulose tris(3,5-dimethylphenylcarbamate)) was used for the enantioseparations of compounds **8** and **10**. Recorded in ethanol, the ECD spectra of the four enantiomer pairs **7**–**10** showed the expected opposite traces for the two atropisomers (Supplementary Materials, Figures S21–S24).

#### 2.2.3. Absolute Configuration Assignment of **7**–**10**

The absolute configuration of HPLC-separated enantiomers of bipyridines **1** and **2** was previously assigned through XRD analysis by using the anomalous dispersion of heavy atoms [48,59]. However, for the enantiomers of bipyridines **3**–**5**, which could not be crystallized, the absolute configuration was determined by electronic circular dichroism (ECD) coupled with time-dependent density functional theory (TD-DFT) calculations [59]. Analogously, the absolute configurations assignment of enantiomers of the newly synthesized bipyridines **7**–**10** was performed by comparing the measured ECD spectra with the ones calculated by TD-DFT. The calculation of the ECD spectra was performed considering all the possible conformers arising from the rotation of the substituent in 2′-position with respect to the pyridine ring [78] (see Supplementary Materials, S6 for details). For each compound, except a systematic shift of about 15 nm between the measured and calculated spectra [50,78], a very good match was obtained allowing unambiguous absolute configuration assignment of all enantiomers (Supplementary Materials, Table S10 and Figures S25–S28).

### 2.3. Biological Evaluation of Compounds ***7–10*** and Structure-Activity Relationships 

The enantiomers of compounds **7**–**10** were tested by using the acid-mediated TTR FF assays described above, in order to evaluate their capability to inhibit fibrillogenesis as soluble TTR is treated with acidic medium. WT- and the mutants Y78F- [8], and V30M-TTR [7] were used as assay proteins, whose stability order is WT > V30M > Y78F [7,8]. The results are reported in Table 2.

Among the three TTR forms, the FF% values were higher against V30M and Y78F than WT-TTR. On the basis of the turbidimetric assay results, the inhibition activity trend was shown to be **9** > **8** > **10** > **7**, with few exceptions. Again, the (*M*) enantiomers were found to be more active than the (*P*)-enantiomers for compounds **9** and **10**, whereas the (*P*)-enantiomers proved to be more effective for **7** and **8**. For both enantiomers of compound **9**, amyloid fibrillogenesis was significantly suppressed on WT-TTR at 7.2 μM of inhibitor concentration (FF% 16 and 17). Both (*M*)-**9** and (*P*)-**9** were also tested at different concentrations ranging from 0 to 15 μM using diflunisal for comparison (Supplementary Information, Figure S29). As reported for other known TTR stabilizers, compound **9** dose-dependently inhibited WT-TTR amyloidogenesis.

**Figure 7:**
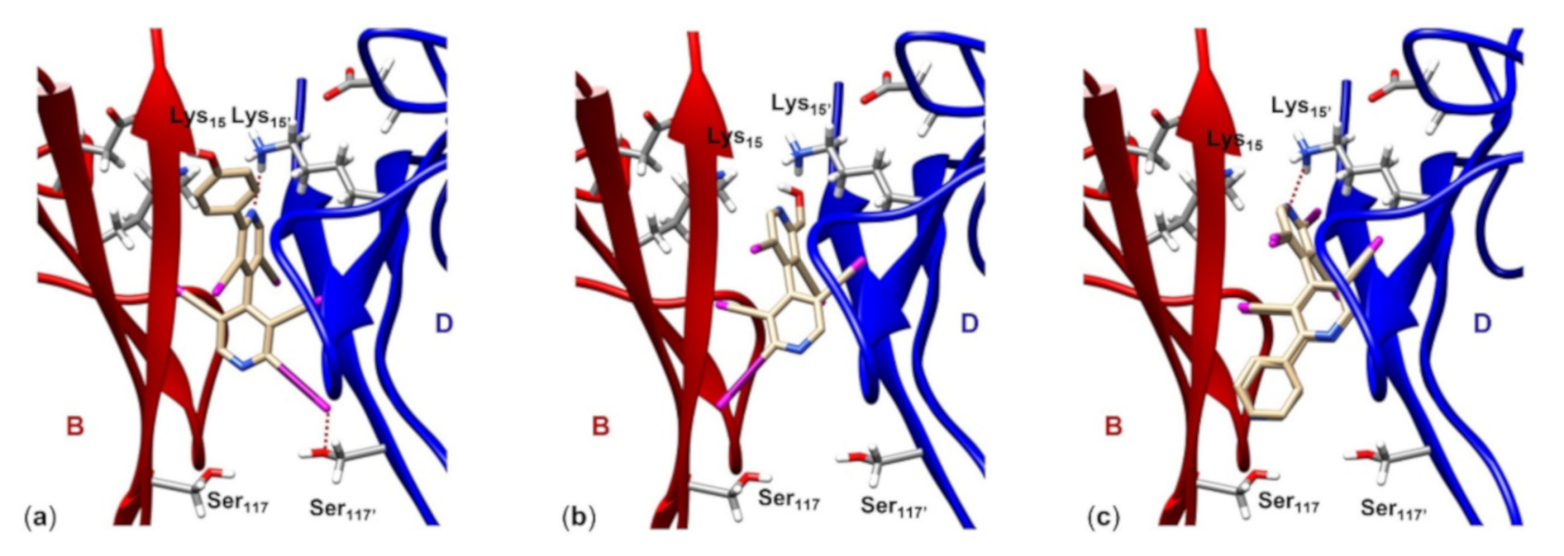
Docking modes into the T_4_ binding pocket (BD) for the (*M*)-enantiomers of **9** (**a**), **10** (**b**) and the pair **7**/**8** (superimposed structures) (**c**).

Structure-activity relationships revealed that a HB centre on one pyridyl ring contributes to increase the inhibition activity, moderately for compounds **8** and **10**, more for compound **9**. In compound **7**, the presence of the hydrophobic phenyl, as distinctive substituent, appears to be detrimental for the inhibition activity. Molecular docking carried out targeting the T_4_ pocket showed that compounds **7**–**10** could be accommodated in the hormone-binding site. Focusing on the four (*M*)-enantiomers, the following observations emerged (Figure 7):

(i) Compound (*M*)-**9** (Figure 7a) fully occupies the T_4_ pocket (molecular length 13.2 Å, *V*_S_ isosurface enclosed volume 367.0 Å^3^), where the 4-hydroxyphenyl group is oriented toward B:Thr_106_ and B:Val_121_ (hBP_1_) and the four chlorines at the 3,3,5,5′-positions of the heteroaromatic ring anchor the residues B:Ala_108_ and D:Ala_108’_ (hBP_2_). Moreover, the *N*’_pyr_ (*V*_S,min_ = -128.3 kJ/mol) forms a HB with the ε-ammonium group of D:Lys_15’_ (2.6 Å), and 2-iodine (*V*_S,max_ = 129.8 kJ/mol) showed a short contact with the side chain oxygen of D:Ser_117’’_ (3.2 Å) (hBP_3_). Interestingly, the comparison of the respective *V*_S_ isosurface evidences for compound **9** a shape similarity to both T_4_ and tafamidis (Supplementary Information, Figure S1);
(ii) Compound **10** (Figure 7b) is the smallest molecule of the series with a molecular length of about 9.80 Å and a volume of 303.1 Å^3^, therefore it is not able to fully occupy the T_4_ pocket, 2-iodine showing a longer contact with B:Ser_117_ (4.4 Å) despite a positive *V*_S,max_ value (141.6 kJ/mol). Moreover, the best conformation of compound **10** is characterized by an intramolecular HB involving the hydroxyl group and N’_pyr_ (*V*_S,min_ = −29.12 kJ/mol). The latter is therefore less prone to interact with the side chain of D:Lys_15’_;
(iii) For both compounds **7** and **8** (Figure 7c), reverse binding modes were found by molecular docking, with the N_pyr_ interacting with D:Lys_15’_ in all cases. The basic difference between the two compounds, which could justify the different inhibition activity, lies in the presence of a N_pyr_, as a HB acceptor, at the 2′-position of compound **8** which makes possible an interaction with B:Ser_117_, thus anchoring the two monomers B and D.

## 3. Materials and Methods

### 3.1. Chemistry

#### 3.1.1. General Information

Proton (^1^H-NMR) and carbon (^13^C-NMR) nuclear magnetic resonance spectra were recorded on Bruker Avance III instruments operating at 500 MHz (Bruker Corporation, Billerica, MA, USA). The chemical shifts are given in parts per million (ppm) on the delta scale. The solvent peak was used as reference values for ^1^H-NMR (CDCl_3_ = 7.26 ppm) and for ^13^C-NMR (CDCl_3_ = 77.16 ppm). Data are presented as follows: chemical shift, multiplicity (s= singlet, d= doublet, t= triplet, q= quartet, quint= quintet, m= multiplet, b= broad), integration, and coupling constants (*J*/Hz). High-resolution mass spectra (HRMS) data were recorded on a Bruker micrOTOF spectrometer equipped with an orthogonal electrospray interface (ESI). Melting points were measured on Stuart SMP3 apparatus from Cole Parmer (London, UK) and are uncorrected. Analytical thin layer chromatography (TLC plates from Merck KGaA, Darmstadt, Germany) was carried out on silica gel 60 F254 plates with visualization by ultraviolet light. Reagents and solvents were purified using standard means. Tetrahydrofuran (THF) was distilled from sodium metal/benzophenone and stored under an argon atmosphere. Anhydrous reactions were carried out in flame-dried glassware and under an argon atmosphere. 2,2′,3,3′,5,5′-Hexaiodo-4,4′-bipyridine (**1**), 2,2′,3,3′,5,5′-hexachloro- 4,4′-bipyridine (**2**), 3,3′,5,5′-tetrachloro-2,2′-diiodo-4,4′-bipyridine (**3**), 2,2′,5,5′-tetrachloro-3,3′- diiodo-4,4′-bipyridine (**4**), 2,2′,3,3′-tetrachloro-5,5′-diiodo-4,4′-bipyridine (**5**) and 3,3′,5,5′-tetra- chloro-2,2′-dibromo-4,4′-bipyridine (**12**) were prepared according to the literature [48]. All other chemicals were used as received. CD spectra were recorded on a J-810 instrument from Jasco (JASCO International Co. Ltd., Tokyo, Japan) at room temperature using 0.15–0.30 mM samples in ethanol and a 1 mm quartz cell, with the following conditions: 100 nm/min scanning speed, 1 nm data pitch, 4.0 nm bandwidth, 1 s response time.

#### 3.1.2. Syntheses of 3,3′,5,5′-Tetrachloro-2,2′-diiodo-4,4′-bipyridine (**3**) and 3,3′,5,5′-tetrachloro-2-iodo- 4,4′-bipyridine (**6**)

In a dry Schlenk tube were placed 2,2′-dibromo-tetrahalo-4,4′-bipyridine (800 mg, 1.77 mmol), NaI (1.06 g, 7.08 mmol), CuI (50 mg, 0.35 mmol), and (*rac*)-*trans*-*N*,*N*-dimethyl- cyclohexane-1,2-diamine (64 mg, 0.71 mmol). The Schlenk tube was evacuated and filled with argon before addition of degassed dioxane (5 mL). The mixture was heated at 120 °C for 60 h. After cooling to room temperature, NH_4_OH (7 mL) and H_2_O (8 mL) were added. Then, the product was extracted with dichloromethane (4 × 40 mL). After drying over Na_2_SO_4_, filtration, and concentration, the mixture was purified by chromatography on silica gel (pentane/dichloromethane 7/3) to give bipyridine **3** [48] (white powder, 733 mg, 76%, R_f_ = 0.27) and bipyridine **6** (white powder, 126 mg, 17%, R_f_ = 0.15). Mp: 126–127 °C; ^1^H-NMR (500 MHz, CDCl_3_) δ 8.67 (s, 2H), 8.46 (s, 1H). ^13^C-NMR (126 MHz, CDCl_3_) δ 148.0; 147.8; 140.6; 140.3; 137.3; 131.2; 130.9; 119.5. HRMS (ESI-TOF) [M + H]^+^ *m*/*z*: Calcd. for C_10_H_3_Cl_4_IN_2_ 418.8168, found: 418.8152.

#### 3.1.3. General Procedure for the Synthesis of Bipyridines **7**, **8** and **14**

In a dry Schlenk tube were placed bipyridine **6** (0.28–0.72 mmol) and tetrakis(triphenylphosphine)palladium (10 mol%). The Schlenk tube was evacuated and filled with argon before addition of degassed toluene (2–5 mL). Then a boronic acid solution (1 eq.) in methanol (0.5–1.0 mL) was added and after 10 min of stirring, at room temperature, a sodium carbonate solution (2 eq.) in distilled water (0.5–1.0 mL) was added. The mixture was heated at 110 °C for 20 h. After cooling to room temperature, brine (10 mL) was added. Then, the product was extracted with dichloromethane (3 × 15 mL). After drying over Na_2_SO_4_, filtration, and concentration, the products were purified by chromatography on silica gel. 

##### 3,3′,5,5′-Tetrachloro-2-iodo-2′-phenyl-4,4′-bipyridine (7) 

Compound **7** was obtained as colorless solid (85 mg, 62%) using the following conditions: **6** (150 mg, 0.28 mmol), tetrakis(triphenylphosphine)palladium (32 mg, 0.028 mmol), phenylboronic acid (34 mg, 0.28 mmol), sodium carbonate (59 mg, 0.55 mmol). Chromatography on silica gel (cyclohexane/ethyl acetate 95/5). Mp: 105–106 °C; ^1^H-NMR (500 MHz, CDCl_3_) δ 8.76 (s, 1H), 8.49 (s, 1H), 7.78–7.71 (m, 2H), 7.53–7.46 (m, 3H). ^13^C-NMR (126 MHz, CDCl_3_) δ 156.2, 147.8, 147.5, 141.9, 141.3, 137.3, 137.1, 131.2, 129.6, 129.5, 129.1, 128.7, 128.4, 119.5. HRMS (ESI-TOF) [M + H]^+^ *m*/*z*: Calcd. for C_16_H_8_Cl_4_N_2_ 495.8481, found: 494.8509.

##### 3,3′,5,5′-Tetrachloro-2-iodo-2′-(4-pyridyl)-4,4′-bipyridine (8)

Compound **8** was obtained as a viscous wax (49 mg, approx. 75% purity according to ^1^H-NMR after chromatography on silica gel with eluent: cyclohexane/ethyl acetate/triethylamine 85/10/5) using the following conditions: **6** (150 mg, 0.28 mmol), tetrakis(triphenylphosphine)palladium (22 mg, 0.019 mmol), 4-pyridinylboronic acid (35 mg, 0.29 mmol), sodium carbonate (59 mg, 0.55 mmol). A pure sample (13.3 mg, 10% yield) was obtained after HPLC purification on a Chiralpak IA column (*n*-hexane/2-propanol 90:10, 0.8 mL/min). ^1^H-NMR (500 MHz, CDCl_3_) δ 8.79 (m, 3H), 8.50 (s, 1H), 7.71 (d, *J* = 5.0 Hz, 2H). ^13^C-NMR (126 MHz, CDCl_3_) δ 153.3, 150.1, 148.0, 147.9, 144.6, 142.4, 140.8, 137.2, 131.1, 130.7, 129.1, 124.0, 119.5. HRMS (ESI-TOF) [M + H]^+^ *m*/*z*: Calcd. for C_15_H_7_Cl_4_IN_3_ 495.8433, found: 494.8400.

##### 3,3′,5,5′-Tetrachloro-2-iodo-2′-(4-((tert-butyldimethylsilyl)oxy)phenyl)-4,4′-bipyridine (14) 

Compound **14** was obtained as viscous oil (199 mg, 44%) using the following conditions: **6** (324 mg, 0.72 mmol), tetrakis(triphenylphosphine)palladium (58 mg, 0.0150 mmol), 4-(*tert*-butyl- dimethylsilyloxy)-phenylboronic acid (190 mg, 0.753 mmol), sodium carbonate (152 mg, 1.43 mmol). Chromatography on silica gel (cyclohexane/ethyl acetate 95/5). ^1^H-NMR (500 MHz, CDCl_3_) δ 8.72 (s, 1*H*), 8.48 (s, 1*H*), 7.68 (d, *J* = 8.7 Hz, 2H), 6.94 (d, *J* = 8.7 Hz, 2H), 1.00 (s, 9H), 0.24 (s, 6H). ^13^C-NMR (126 MHz, CDCl_3_) δ 157.1; 155.8; 147.8; 147.3; 141.9; 141.5; 137.3; 131.3; 131.1; 130.1; 128.5; 128.4; 120.0; 119.5; 25.8; 18.4; −4.2. HRMS (ESI-TOF) [M + H]^+^ *m*/*z*: Calcd. for C_22_H_22_Cl_4_IN_2_OSi 624.9275, found: 624.9295.

#### 3.1.4. 3,3′,5,5′-Tetrachloro-2-iodo-2′-(4-hydroxyphenyl)-4,4′-bipyridine (**9**)

To a stirred solution of bipyridine **14** (70 mg, 0.11 mmol) in THF (3 mL), under argon and at 0 °C, a TBAF solution (0.016 mL, 1M in THF) was slowly added. After 30 min stirring at 0 °C, the reaction was quenched with NH_4_Cl and extracted with dichloromethane (3 × 10 mL). After drying over Na_2_SO_4_, filtration, and concentration, the product was purified by chromatography on silica gel (cyclohexane/ethyl acetate 85/15) to give **9** as a viscous wax (50 mg, 87%). ^1^H-NMR (500 MHz, CDCl_3_) δ 8.72 (s, 1H), 8.48 (s, 1H), 7.69 (d, *J* = 8.7 Hz, 2H), 6.93 (d, *J* = 8.7 Hz, 2H), 5.24 (s, 1H). ^13^C- NMR (126 MHz, CDCl_3_) δ 156.9; 155.6; 147.8; 147.3; 142.0; 141.4; 137.3; 131.4; 131.2; 129.6; 128.6; 128.4; 119.5; 115.3. HRMS (ESI-TOF) [M + H]^+^ *m*/*z*: Calcd. for C_22_H_22_Cl_4_IN_2_OSi 510.8430, found: 510.8449.

#### 3.1.5. Synthesis of 3,3**’**,5,5**’**-Tetrachloro-2-iodo-2**’**-hydroxymethyl-4,4**’**-bipyridine (**10**)

Bipyridine **3** (0.458 mmol, 250 mg) was dissolved in toluene (5 mL) and the solution was cooled to −78 °C. *n*-BuLi (1.2 M, 0.458 mmol, 0.38 mL) was added dropwise and the mixture was stirred at −78 °C for 1 h. DMF (0.916 mmol, 71 μL) was added and stirring was continued at the same temperature for 1 h. NaBH_4_ (1.374 mmol, 52.2 mg) was added followed by methanol (0.5 mL), the cooling bath was removed and stirring was maintained for 1 h. The mixture was hydrolysed at 0 °C and extracted with ethyl acetate. After drying over Na_2_SO_4_, filtration and evaporation, the residue was purified twice by chromatography on silica gel (cyclohexane/ethyl acetate 9/1) to give bipyridine **10** as colourless wax (66 mg, 18%). ^1^H-NMR (500 MHz, CDCl_3_) δ 8.67 (s, 1H); 8.48 (s, 1H); 4.85 (s, 2H), 3.98 (bs, 1H). ^13^C-NMR (126 MHz, CDCl_3_) δ 155.3; 147.8; 146.2; 141.3; 140.3; 137.3; 131.1; 129.6; 128.0; 119.5; 61.9. HRMS (ESI-TOF) [M + H]^+^ *m*/*z*: Calcd. for C_11_H_5_Cl_4_IN_2_O 448.8273, found: 448.8281.

#### 3.1.6. Multimilligram Enantioseparation of **7**–**10**

An Agilent Technologies (Waldbronn, Germany) 1100 Series HPLC system (high-pressure binary gradient system equipped with a diode-array detector operating at multiple wavelengths (220, 254, 280, 360 nm), a programmable autosampler with a 20 L loop, and a thermostatted column compartment) was employed for analytical and multimilligram separations. Data acquisition and analysis were carried out with Agilent Technologies ChemStation Version B.04.03 chromatographic data software. The UV absorbance is reported as milliabsorbance units (mAU). Chiralcel OD-H (cellulose tris-3,5-dimethylphenylcarbamate), and Chiralpak IA (amylose tris-3,5-dimethyl- phenylcarbamate) (5 μm; Chiral Technologies Europe, Illkirch, France) were used as analytical (250 × 4.6 mm) chiral columns. HPLC-grade *n*-hexane, 2-propanol, and methanol were purchased and used as received. Analyses were performed in isocratic mode. Chromatographic separations were performed at 22 °C. After multimilligram enantioseparation, the collected fractions were analyzed under the same conditions used for separation to determine their enantiomeric excess (ee).

### 3.2. Biological Details

#### 3.2.1. TTR Expression and Purification

Recombinant WT-hTTR and mutants Y78F-, V30M-hTTR were produced using a pET expression system (GeneCust, Boynes, France) as essentially described by Dolado and co-workers [77]. All the hTTR proteins were expressed in *Escherichia coli* BL21-(DE3) cells harboring the corresponding plasmid. Expression cultures were grown in LB medium supplemented with 100 μg/mL Ampicillin at 36 °C to an optical density (at 600 nm) of 0.5. Protein expression was induced by addition of 0.4 mM IPTG for 5 h, then bacteria were harvested by centrifugation (5500 *g* for 20 min), washed with PBS and stored at −80 °C. Bacterial pellet was resuspended in 20 mM Tris-HCl pH 7.5, 1 mM ethylenediaminetetraacetic acid (EDTA), 100 μM PMSF. After enzymatic lysis with 1 mg/mL lysozyme, cells were treated with 2.5 μg/mL deoxyribonuclease I, 10 mM MgCl_2_, 50 mM NaCl, centrifuged and the clear supernatant collected. Proteins were fractionated by ammonium sulfate precipitation between 55 and 85% saturation. The precipitate was dissolved in 20 mM Tris-HCl pH 7.2, dialyzed against the same buffer and then fractionated by anion exchange chromatography on a Q-Sepharose column with a 0–0.6 M NaCl gradient in 20 mM Tris pH 7.2. A final step on Superdex 75 was conducted in 10 mM potassium phosphate pH 7.6 containing 100 mM KCl. The purity of protein fractions was assessed by standard SDS-PAGE analysis. Pure proteins equilibrated in buffer phosphate 10 mM pH 7.6, 100 mM KCl, 1 mM EDTA were stored at −20 °C. The extinction coefficients of WT- and V30M-TTR, 7.76 × 104 M^−1^cm^−1^ [79], and Y78F-TTR, 6.958 × 104 M^−1^cm^−1^ [42], were used to determine the protein concentration by measuring the absorbance at 280 nm.

#### 3.2.2. Acid-mediated TTR Fibril Formation Assay

The assay was conducted as described by Klabunde and co-workers [34]. 150 μL of a 0.4 mg/mL (7.2 μM) stock of WT or mutants hTTR in 10 mM phosphate buffer pH 7.6, containing 100 mM KCl and 1 mM EDTA, were incubated with 1.45 μL of the selected drug (dissolved in DMSO) in an Eppendorf tube at 36 °C. After 30 min, the pH was lowered (up to 4.4 in the case of WT or 5.0 in the case of mutants) by adding 150 μL of acidic sodium acetate buffer 200 mM (pH 4.2 for WT, 4.8 for Y78F and V30M), containing 100 mM KCl and 1 mM EDTA. The mixtures were incubated at 36 °C for 72 h, after which the samples were vortexed and turbidity was measured at 400 nm in quartz cuvette. The final concentration of the protein was 3.6 μM, while drug concentrations was 3.6 or 7.2 μM. Data were expressed as percentage of fibril formation compared to that recorded in absence of drugs defined to be 100%. The percentage of fibril formation was determined by observing turbidity increase at 400 nm. Each measurement was run in triplicate. Both (*M*)-**9** and (*P*)-**9** were also tested at different concentrations ranging from 0 to 15 μM. The assay was conducted as described above using WT-TTR 3.6 μM.

### 3.3. Computationals

#### 3.3.1. Electrostatic Potential Isosurfaces

Conformational search was performed employing the density functional theory (DFT) method with the B3LYP functional and the 6-311G* basis set, and the Spartan’ 10 Version 1.1.0 (Wavefunction Inc., Irvine, CA, USA) program [80]. Geometry optimization and computation of electrostatic potentials isosurfaces and related parameters (*V*_S_ extrema, *V*_S,max_ and *V*_S,min_ values, given in kJ/mol) were performed by using Gaussian 09 (DFT, B3LYP, 6-311G*) (Wallingford, CT, USA) [81]. Search for the exact location of such EP maxima and minima was made through the Multiwfn code [82] and through its module enabling quantitative analyses of molecular surfaces (isovalue 0.002) [83].

#### 3.3.2. In Silico Docking Study

The crystal structure complex TTR (chains ABCD)-T_4_ was released from the RCSB protein databank (PDB ID: 1ICT) (www.rcsb.org) [2]. The crystallographic T_4_ ligands were extracted and hydrogen atoms were added to the TTR structure using ADT module. Computational modelling experiments were carried out on a HP8100 PC and an EXXACT Tensor Workstation TWS-1686525-AMB with OS Ubuntu 18.4 or Windows 10, and Centos, respectively. The atomic charges were assigned using the Gasteiger-Marsili method for the 4,4′-bipyridines and the protein [84]. Binding of the compounds was analysed using MGLTools 1.5.7rc1 [85] and AutoDock 4.2.6 docking programs [68,69]. The structures were docked using the Lamarckian genetic algorithm (LGA). In the case of the blind docking procedure, the LGA was defined through a centred grid, coordinates: x = −7.5, y = −45.5, z = 38.5, with 126 × 126 × 126 grid points in x, y, z dimensions, respectively, spacing 0.550 Å. For the docking focused on the T_4_ binding site, 40 × 40 × 40 grid points in x, y, z dimensions, respectively, spacing 0.375 Å. All 4,4′-bipyridine enantiomers were docked with the pyridyl-pyridyl bond fixed, blocking rotation. The Lamarckian genetic algorithm (LGA) of up to 100 runs was set to the population size of 150 individuals, maximum number of generations and energy evaluations of 27,000 and 25,000,000, respectively. From the estimated free energy of ligand binding (E.F.E.B., ΔG), the estimated inhibition constant (E.I.C., K_i_) for each 4,4′-bipyridine was calculated. K_i_ is calculated by the equation: K_i_ = exp [(ΔG × 1000)/(R × T)] where ΔG is the docking estimated free energy, R (gas constant) = 1.98719 cal/(K × mol) and T = 298.15 K. Graphical representation of the poses derived from the docking calculation was obtained using the software Chimera 1.13.1 [70]. 4,4′-Bipyridines **1**–**10** were constructed by using the standard bond lengths and angles from the fragment database of GaussView 5.0 and optimized with Gaussian 09 (DFT, B3LYP, 6-311G*) [81,86]. The explicit σ-hole (ESH) was used as previously described [51,64,65], in order to account for the charge anisotropy of the electrostatic potential on top of the halogen atoms. On this basis, a massless dummy atom connected to *I* and *Cl* was introduced manually, by using distance and charge values as described by Hobza and co-workers [65]. The parameters used for *Cl* and *I* were 1.0, 1.6 Å, and 0.1, 0.3 units of positive charge for the extra point (ExP), respectively (Supplementary Information, Table S7). The reliability of the docking approach was further verified by extracting the T_4_ ligands from the T_4_ binding pocket of the 1ICT.pdb, 3.00 Å resolution crystal structure, and by considering it as a normal ligand. A massless dummy atom connected to the iodines of T_4_ was introduced manually in order to model the σ-hole, as previously described. After repositioning of T_4_ into the protein, the new T_4_ location was in accord to the original X-ray structure of TTR (Supplementary Information, Figure S3), with only minimal conformational changes (RMSD 0.57 Å), therefore confirming the reliability of the system.

## 4. Conclusions

Unexplored as a substructure for the development of TTR stabilizers, the 4,4′-bipyridyl motif has been proposed and used herein for the development of new potential fibrillogenesis inhibitors. Among the screened derivatives, the 3,3′,5,5′-tetrachloro-2-iodo-4,4′-bipyridyl core emerged as a key chiral scaffold, and several derivatives containing a 2′-substituent were thus prepared by focused/specific synthetic procedures. The enantioseparation of these chiral compounds made available their enantiomers, which were tested against acid-mediated TTR fibrillogenesis. For each compound, low or moderate differences were observed between the enantiomer pairs *M*/*P*. Among all enantiomers, (*M*)-3,3′,5,5′-tetrachloro-2′-(4-hydroxyphenyl)- 2-iodo-4,4′-bipyridine proved to be able to significantly reduce FF of WT-TTR (16% at 7.2 μM of inhibitor concentration). In silico docking studies within the T_4_ pocket were carried out in order to rationalize the binding mode and hypothesize a possible molecular mechanism for the observed inhibition activity. Both experimental and theoretical results evidenced the importance of HB sites located in the distinctive 2′-substituent and the capability of 2-iodine as XB donor. Despite the fact that some issues such as plasma TTR binding selectivity has still to be tackled and that the new structures need optimization, the present study revealed the potentiality of halogenated 4,4′-bipyridines for the development of new TTR amyloidogenesis inhibitors.

## Figures and Tables

**Table 1:** Inhibition of WT- and Y78F-TTR amyloid fibril formation under acidic denaturation condition in the presence of diflunisal, pure enantiomers of derivatives **1**–**5**, and achiral **6**.

Compound	FF% ^1^ (WT-TTR)		FF% ^1^ (Y78F-TTR)	
	7.2 μM	3.6 μM	7.2 μM	3.6 μM
Diflunisal	6	18	18	43
(*M*)-**1**	99	100	100	100
(*P*)-**1**	100	100	100	100
(*M*)-**2**	93	98	89	94
(*P*)-**2**	95	97	87	93
(*M*)-**3**	94	94	72	62
(*P*)-**3**	96	100	85	87
(*M*)-**4**	96	97	87	91
(*P*)-**4**	96	99	95	95
(*M*)-**5**	90	97	85	91
(*P*)-**5**	98	98	100	94
**6**	91	95	73	82

^1^ Fibril formation (FF)% represents the extent of amyloidogenic TTR FF in the presence of inhibitors (7.2 μM or 3.6 μM inhibitor, 3.6 μM TTR, pH 4.4 (WT) or 5.0 (Y78F), 36 °C, 72 h), compared to untreated TTR (100% fibril formation). The experiments were performed in triplicate and the average values are reported in the Table. The standard deviations were in the range 5–10.

**Table 2:** Inhibition of WT-, Y78F-, and V30M-TTR amyloid fibril formation under acidic denaturation condition in the presence of diflunisal, and pure enantiomers of derivatives **7**–**10**.

Compound	FF% ^1^ (WT-TTR)		FF% ^1^ (Y78F-TTR)		FF%^1^ (V30M-TTR)	
	7.2 μM	3.6 μM	7.2 μM	3.6 μM	7.2 μM	3.6 μM
Diflunisal	6	18	18	43	36	33
(*M*)-**7**	96	100	82	84	nd	nd
(*P*)-**7**	88	95	71	88	nd	nd
(*M*)-**8**	58	72	79	82	50	58
(*P*)-**8**	42	67	51	67	38	43
(*M*)-**9**	16	49	44	71	38	40
(*P*)-**9**	17	49	44	72	39	41
(*M*)-**10**	62	79	80	88	59	75
(*P*)-**10**	80	89	85	91	67	72

^1^ Fibril formation (FF)% represents the extent of amyloidogenic TTR FF in the presence of inhibitors (7.2 μM or 3.6 μM inhibitor, 3.6 μM TTR, pH 4.4 (WT) or 5.0 (V30M and Y78F), 36 °C, 72 h), compared to untreated TTR (100% fibril formation). The experiments were performed in triplicate and the average values are reported in the Table. The standard deviations were in the range 5–10; nd, not determined.

## Supplementary Materials

The following are available online: Figure S1: *V*_S_ molecular isosurfaces (0.002 au) calculated for (*P*) and (*M*) enantiomers of **9**, T_4_, and Tafamidis, Figure S2: Blind docking poses of compounds (*M*)-**3** and **6** on the whole TTR, Figure S3: Comparison of the docked pose of T_4_ into TTR (BD:T_4_ binding pocket) with the crystallographic structure of T_4_ (released from PDB ID: 1ICT), Figure S4: Linear regression analysis describing the relationships between FF% and calculated EFEB by docking in the T_4_ pocket (BD), Figures S5-S16: NMR spectra, Figures S17-S20: HPLC chromatograms, Figures S21-S24: ECD spectra, Figures S25-S28: comparison of calculated and experimental ECD spectra, Figure S29: Inhibition of WT-TTR in the presence of (*M*)-**9** and (*P*)-**9** tested at different concentrations, Table S1: Calculated *V*_S_ on a 0.002 au isosurface and molecular geometrical parameters for polyhalogenated 4,4′-bipyridines **1**–**6**, Table S2: Calculated *V*_S_ on a 0.002 au isosurface and molecular geometrical parameters for Tafamidis and Thyroxyne (T_4_) (from crystal structure, PDB ID:1ICT), Table S3: Calculated distribution, pattern and properties of conformations A and B of 4,4′-bipyridines **7**–**9**, Table S4: Calculated distribution, pattern and properties of conformations A-F of 4,4′-bipyridine **10**, Table S5: Calculated *V*_S_ on a 0.002 au isosurface and molecular geometrical parameters for conformations A and B of 2′-aryl-3,3′,5,5′-tetrachloro-2-iodo-4,4′-bipyridines **7**–**9**, Table S6: Calculated *V*_S_ on a 0.002 au isosurface and molecular geometrical parameters for conformations A-F of 4,4′-bipyridine **10**, Table S7: Parameters used for the extra point (ExP) of charge (X = Cl, I), Table S8: Blind docking resultsa targeting poses found in the T_4_ binding pockets, Table S9: Docking results for 4,4′-bipyridines **1**–**6** in the T_4_ binding pocket, Table S10: Optimized multimilligram enantioseparation of 4,4′-bipyridines **7**–**10**.
