# Supplementary material for: Rational Design, Synthesis, Characterization and Evaluation of Iodinated 4,4′-Bipyridines as New Transthyretin Fibrillogenesis Inhibitors
Source: Molecules. 2020 May 8;25(9):2213. doi: 10.3390/molecules25092213 (PMC7248964; doi:10.3390/molecules25092213)
Supplement: Supplementary file 1 [file molecules-25-02213-s001.pdf]

Supplementary Information

Rational design, synthesis, characterization and evaluation of iodinated 4,4’-bipyridines as new transthyretin fibrillogenesis inhibitors

Alessandro Dessì,[a] Paola Peluso,[a,]* Roberto Dallocchio,[a] Robin Weiss,[b] Giuseppina Andreotti,[c] Mariateresa Allocca,[c,d] Emmanuel Aubert,[e] Patrick Pale,[b] Victor Mamane,[b,]* and Sergio Cossu[f]

[a] Istituto di Chimica Biomolecolare ICB-CNR, Sede secondaria di Sassari, Traversa La Crucca 3, Regione Baldinca, 07100 Li Punti - Sassari, Italy
[b] Institut de Chimie de Strasbourg, UMR CNR 7177, Equipe LASYROC, 1 rue Blaise Pascal, 67008 Strasbourg Cedex, France
[c] Istituto di Chimica Biomolecolare ICB-CNR, Via Campi Flegrei 34, 80078 Pozzuoli (NA), Italy
[d] Università degli Studi della Campania “Luigi Vanvitelli”, Italy
[e] Cristallographie, Résonance Magnétique et Modélisations (CRM2), UMR CNRS 7036, Université de Lorraine, Bd des Aiguillettes, 54506 Vandoeuvre-les-Nancy, France
[f] Dipartimento di Scienze Molecolari e Nanosistemi DSMN, Università Ca’ Foscari Venezia, Via Torino 155, 30172 Mestre Venezia, Italy

Table of contents

S1. Computation of V_S on a 0.002 au isosurface, and related parameters.

Calculation of conformer distribution (vacuum) for compounds **7-10** 2

S2. Molecular docking 7

S3. NMR spectra 11

S4. HPLC enantioseparations 17

S5. Electronic circular dicroism (ECD) spectra of pure enantiomers of compounds **7-10** 22

S6. Absolute configuration assignment 24

S7. Inhibition of WT-TTR in the presence of (*M*)-**9** and (*P*)-**9** tested at different concentrations 28

S1. Computation of V_S on a 0.002 au isosurface, and related parameters. Calculation of conformer distribution (vacuum) for compounds 7-10

Table S1

Calculated V_S[a] on a 0.002 au isosurface and molecular geometrical parameters[b] for polyhalogenated 4,4'-bipyridines **1-6**: V_S (kJ/mol), surface volume (Å^3), surface area (Å^2), length (Å), width (Å).

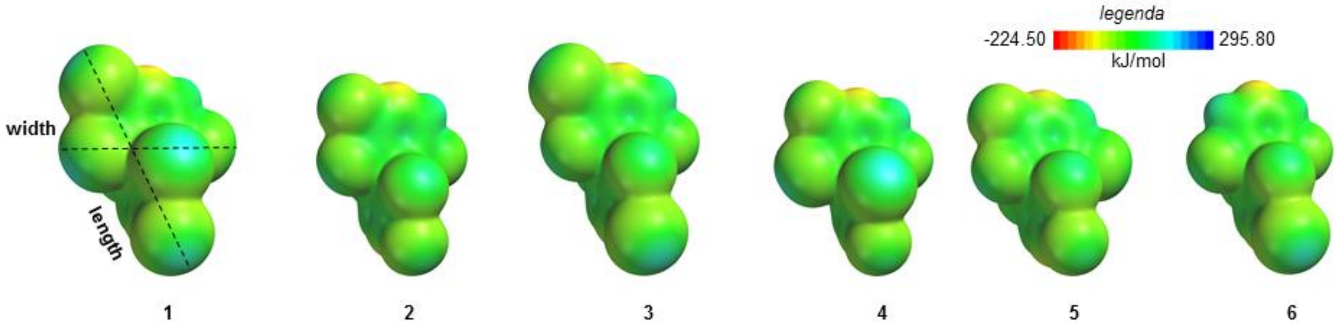

	polyhalogenated 4,4'-bipyridine					
Descriptor	**1**	**2**	**3**	**4**	**5**	**6**
			$V_{S,min}$			
N1	-121.66	-118.39	-117.07	-122.39	-120.87	-121.34
N1'	-121.72	-118.31	-117.19	-122.43	-120.87	-127.35
			$V_{S,max}$			
Pyr	72.30;73.32	96.37;96.58	89.13;89.43	85.66;87.46	88.23;89.22	82.36;82.66
Pyr'	72.06;73.57	95.97;96.43	88.96;89.36	85.33;87.18	87.81;88.95	71.01;71.10
2-X	133.42 (I)	75.00 (Cl)	140.83 (I)	71.08 (Cl)	72.93 (Cl)	136.83 (I)
2'-X	133.53 (I)	75.04 (Cl)	140.93 (I)	70.96 (Cl)	72.97 (Cl)	--
3-X	157.39 (I)	101.00 (Cl)	96.38 (Cl)	166.62 (I)	96.57 (Cl)	92.06 (Cl)
3'-X	157.22 (I)	101.09 (Cl)	96.18 (Cl)	166.71 (I)	96.56 (Cl)	88.36 (Cl)
5-X	158.79 (I)	101.59 (Cl)	98.81 (Cl)	96.30 (Cl)	167.02 (I)	94.38 (Cl)
5'-X	158.70 (I)	101.83 (Cl)	98.79 (Cl)	96.44 (Cl)	167.06 (I)	88.48 (Cl)
			molecular geometrical parameters			
Volume	373.43	277.07	309.37	309.02	309.54	275.77
Area	336.11	269.89	296.41	289.75	292.52	267.82
Length	9.13	8.51	9.26	8.62	8.49	8.29
Width	6.17	5.45	5.45	5.82	5.83	5.47

[a] Computation of V_S on a 0.002 au isosurface and related parameters were performed by using Gaussian 09 (DFT, B3LYP, 6-311G*) (Wallingford, CT 06492, USA). Search for the exact location of $V_{S,min}$ and $V_{S,max}$ was made through the Multiwfn code, and through its module enabling quantitative analyses of molecular surfaces (isovalue 0.002). [b] Spartan' 10 Version 1.1.0 graphic interface.

Table S2

Calculated V_S[a] on a 0.002 au isosurface and molecular geometrical parameters[b] for Tafamidis and Thyroxyne (T_4) (from crystal structure, PDB ID:1ICT)[c]: V_S (kJ/mol), surface volume (Å^3), surface area (Å^2), length (Å), width (Å).

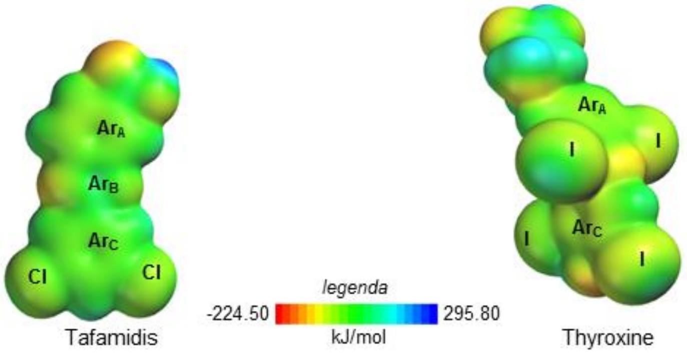

Descriptor	Tafamidis	Descriptor	Thyroxine
	$V_{S,max}$		$V_{S,max}$
Ar (**A**)	19.62;19.77	Ar (**A**)	11.34
Ar (**B**)	98.49;98.88		
Ar (**C**)	36.70;37.05	Ar (**C**)	-5.58;-5.07
$Cl_{\mathbf{C}}$	70.79	$I_{\mathbf{C}}$	109.11
$Cl_{\mathbf{C}}$	74.33	$I_{\mathbf{C}}$	143.05
		$I_{\mathbf{A}}$	122.43
		$I_{\mathbf{A}}$	128.03
	molecular geometrical parameters		*molecular geometrical parameters*
Volume	270.11	Volume	417.74
Area	277.64	Area	402.16
Length	12.40	Length	14.43
Width	5.42	Width	6.11

[a] Computation of V_S on a 0.002 au isosurface and related parameters were performed by using Gaussian 09 (DFT, B3LYP, 6-311G*) (Wallingford, CT 06492, USA). Search for the exact location of $V_{S,min}$ and $V_{S,max}$ was made through the Multiwfn code, and through its module enabling quantitative analyses of molecular surfaces (isovalue 0.002). [b] Spartan' 10 Version 1.1.0 graphic interface.[c] A. Wojtczak, P. Neumann, V. Cody, Acta Cryst. D57 (2001) 957-967

Table S3

Calculated[a] distribution, pattern and properties of conformations A and B of 4,4'-bipyridines **7-9**: energy (au), Boltzmann distribution (%), a-b-c-d and a'-b'-c'-d' dihedral angles (°).

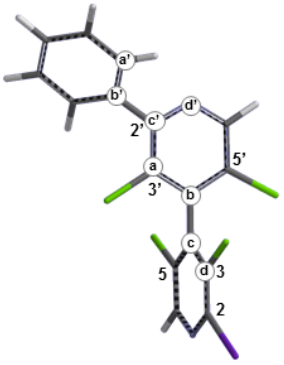

conformation 'A'

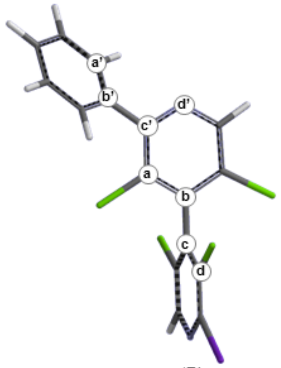

'B'

bipyridine	Conformation	Energy (au)	Boltzmann distribution (%)	Dihedral angles (°)	
				a-b-c-d	a'-b'-c'-d'
**7**	A	-9483.96542	49.5	-95.48	-44.23
	B	-9483.96544	50.5	-91.70	42.25
**8**	A	-9500.00498	49.3	-95.32	-44.29
	B	-9500.00503	50.7	-91.71	42.22
**9**	A	-9559.20517	53.4	-95.29	-36.87
	B	-9559.20504	46.6	-93.96	38.31

[a] Spartan' 10 Version 1.1.0 (Wavefunction Inc., Irvine, CA), DFT/B3LYP/6-311G*.

Table S4

Calculated[a] distribution, pattern and properties of conformations A-F of 4,4'-bipyridine **10**: energy (au), Boltzmann distribution (%), a-b-c-d and a'-b'-c'-d' dihedral angles (°).

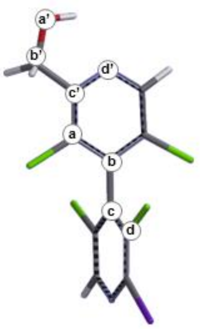

A-B-C

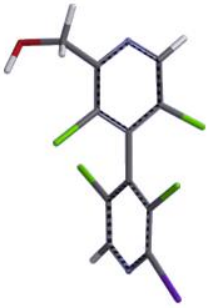

D

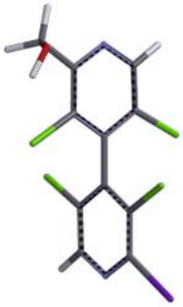

E

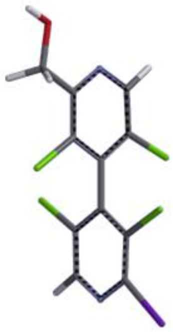

F

Conformation	Energy (au)	Boltzmann distribution (%)	Dihedral angles (°)	
			a-b-c-d	a'-b'-c'-d'
A	-9367.42227	47.3	-89.07	-0.06
B	-9367.42178	28.2	-96.20	2.07
C	-9367.42164	24.2	-86.15	2.85
D	-9367.41699	0.2	-87.58	112.16
E	-9367.41697	0.2	-93.92	-111.62
F	-9367.41264	0.0	-87.69	1.12

[a] Spartan' 10 Version 1.1.0 (Wavefunction Inc., Irvine, CA), DFT/B3LYP/6-311G*.

Table S5

Calculated V_S[a] on a 0.002 au isosurface and molecular geometrical parameters[b] for conformations A and B of 2'-aryl-3,3',5,5'-tetrachloro-2-iodo-4,4'-bipyridines **7**-**9**: V_S (kJ/mol), surface volume (Å^3), surface area (Å^2), length (Å), width (Å).

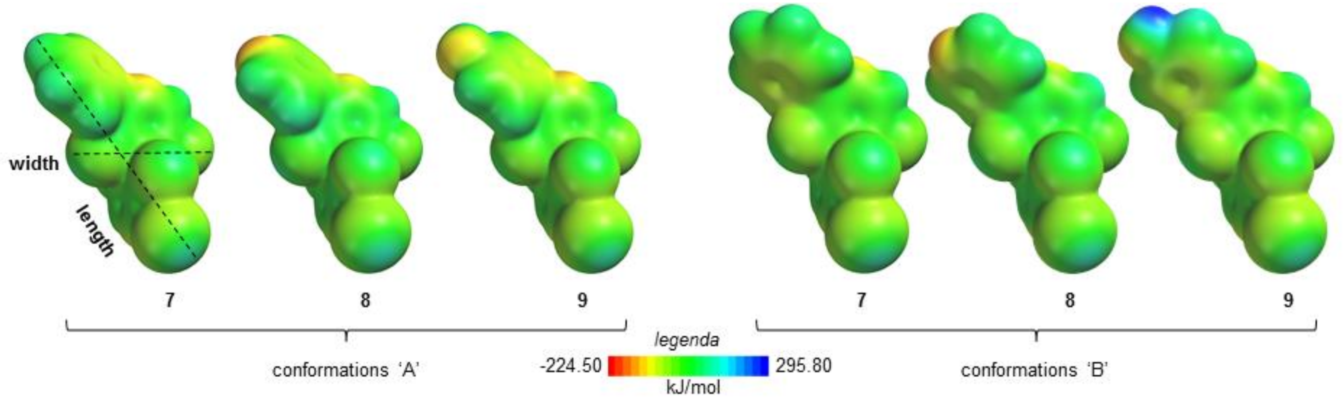

	**7**		**8**		**9**	
Descriptor	A	B	A	B	A	B
			$V_{S,min}$			
N1	-125.92	-125.99	-118.03	-118.13	-128.57	-128.66
N1'	-130.81	-131.06	-111.87	-112.30	-128.33	-128.32
N_{pyr} (**8**)	--	--	-172.86	-172.98	--	--
<u>O</u>H (**9**)	--	--	--	--	-110.62	-110.46
			$V_{S,max}$			
Pyr	75.08;75.55	75.13;75.56	86.71;86.80	86.13;86.39	71.91;72.37	71.59;71.65
Pyr'	60.25;61.02	60.07;61.32	79.95;80.75	80.13;81.13	53.43;54.36	53.88;55.43
2-I	132.78	131.97	140.56	139.85	130.12	129.82
2'-Ar	-35.70;-40.09	-36.15;-39.53	4.56;7.75	5.12;7.55	-26.85;-30.92	-27.46;-30.78
O<u>H</u> (**9**)	--	--	--	--	296.14	295.80
3-Cl	88.39	86.16	98.37	96.20	84.76	83.83
3'-Cl	79.01	78.89	93.65	93.61	75.07	74.63
5-Cl	88.57	90.67	98.53	100.24	86.30	87.35
5'-Cl	82.29	82.06	95.24	94.95	78.39	78.25
			molecular geometrical parameters			
Volume	358.28	358.28	353.10	353.11	366.97	366.96
Area	340.63	340.76	336.43	336.57	349.24	349.22
Length	12.07	12.21	11.13	11.23	13.12	13.02
Width	5.44	5.44	5.44	5.44	5.44	5.44

[a] Computation of V_S on a 0.002 au isosurface and related parameters were performed by using Gaussian 09 (DFT, B3LYP, 6-311G*) (Wallingford, CT 06492, USA). Search for the exact location of $V_{S,min}$ and $V_{S,max}$ was made through the Multiwfn code, and through its module enabling quantitative analyses of molecular surfaces (isovalue 0.002). [b] Spartan' 10 Version 1.1.0 graphic interface.

Table S6
Calculated V_S[a] on a 0.002 au isosurface and molecular geometrical parameters[b] for conformations A-F of 4,4’-bipyridine **10**: V_S (kJ/mol), surface volume (Å^3), surface area (Å^2), length (Å), width (Å).

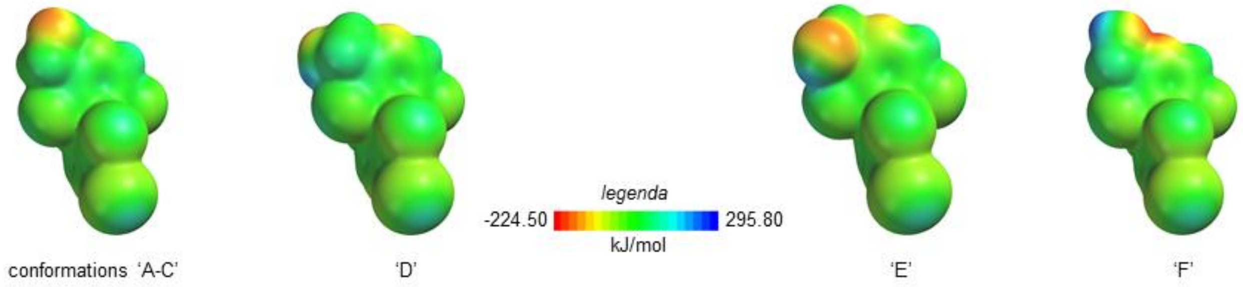

Descriptor	A-C	D	E	F
		$V_{S,min}$		
N1	-116.33	-118.65	-118.94	-124.90
N1’	-29.12	-119.60	-119.71	-224.50
2’-CH$_2$OH	-173.85	-158.94	-159.72	(-224.50)
		$V_{S,max}$		
Pyr	89.45;89.94	85.21;86.96	84.47;86.94	75.91;78.20
Pyr’	84.64;84.80	66.02;74.63	65.88;75.15	56.77;57.11
2-I	141.60	139.35	138.69	133.51
2’-CH$_2$OH	145.47	217.77	216.51	256.70
3-Cl	98.33	96.49	90.77	87.57
3’-Cl	98.34	106.50	106.57	89.70
5-Cl	100.71	93.19	98.43	90.14
5’-Cl	96.41	90.00	89.68	78.22
		molecular geometrical parameters		
EPS_{volume}	303.13	303.80	303.82	303.79
EPS_{area}	292.78	292.86	292.88	293.78
Length	9.80	9.22	9.66	10.46
Width	5.47	5.46	5.46	5.46

[a] Computation of V_S on a 0.002 au isosurface and related parameters were performed by using Gaussian 09 (DFT, B3LYP, 6-311G*) (Wallingford, CT 06492, USA). Search for the exact location of $V_{S,min}$ and $V_{S,max}$ was made through the Multiwfn code, and through its module enabling quantitative analyses of molecular surfaces (isovalue 0.002). [b] Spartan’ 10 Version 1.1.0 graphic interface.

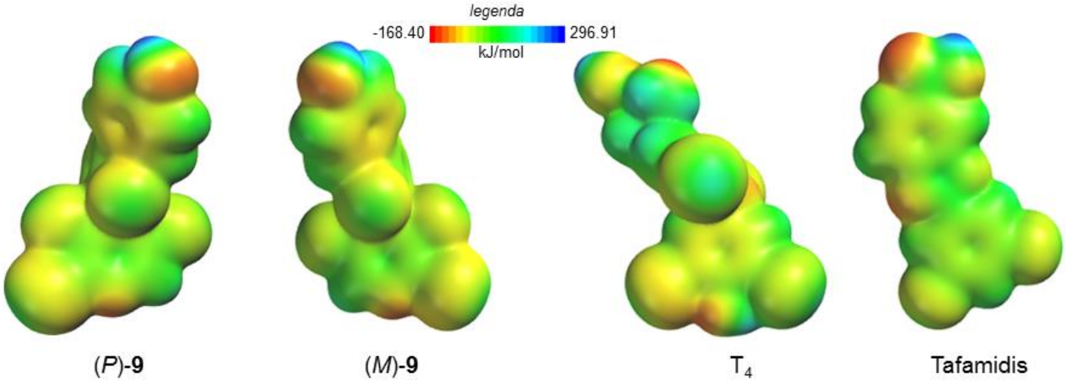

Figure S1. V_S molecular isosurfaces (0.002 au) calculated for (*P*) and (*M*) enantiomers of **9**, T_4, and Tafamidis.

S2. Molecular docking

Table S7
Parameters used for the extra point (ExP) of charge (X = Cl, I).[a]

Mass ExP	0.00 amu
r (ExP)	1.00 Å
ε (ExP)	0.00 Å
r_{eq} (Cl-ExP)	1.00 Å
r_{eq} (I-ExP)	1.60 Å
K_r (X-ExP)	600.0
Θ_{eq} (A-X-ExP)	180.0°
K_θ (A-X-ExP)	150.0
γ (A-A-X-ExP)	0.00°
V_n(A-A-X-ExP)	0.00

[a] a) P. Peluso, V. Mamane, E. Aubert, A. Dessì, R. Dallocchio, A. Dore, P. Pale, S. Cossu, J. Chromatogr. A 1467 (2016) 228–238; b) M.A.A. Ibrahim, J. Mol. Model. 18 (2012) 4625-4638; c) M. Kolar, P. Hobza, K. Bronowska, Chem.Commun. 49 (2013) 981-983.

Table S8
Blind docking results[a] targeting poses found in the T_4 binding pockets.[b]

bipyridine	binding energy range (all poses) [kcal/mol]	T_4 docking score (binding energy [kcal/mol])	Interacting aminoacid residues
M-**1**	-5.43 / -4.73	0	--
P-**1**	-5.00 / -4.63	0	--
M-**2**	-5.45 / -4.36	8% (-5.45)	B:Leu110 B:Thr119 B:Lys15 B:Leu17 D:Ala108 D:Ala109 D:Leu110 D:Ser117 D:Thr119 D:Lys15 D:Leu17
P-**2**	-5.26 / -4.37	12% (-5.26)	B:Leu110 B:Thr119 B:Lys15 B:Leu17 D:Ala108 D:Ala109 D:Leu110 D:Ser117 D:Thr119 D:Lys15 D:Leu17
M-**3**	-6.14 / -4.42	12% (-6.14)	B:Ala109 B:Leu110 B:Ser117 B:Thr119 B:Lys15 B:Leu17 D:Thr106 D:Ala108 D:Leu110 D:Thr119 D:Lys15 D:Leu17
		5% (-6.11)	B:Leu110 B:Thr119 B:Val121 B:Leu17 D:Ala108 D:Leu110 D:Ser117 D:Thr119 D:Lys15 D:Leu17
		4% (-5.44)	A:Leu110 A:Ser117 A:Thr119 A:Lys15 A:Leu17 C:Thr106 C:Ala108 C:Ala109 C:Leu110 C:Thr119 C:Val121 C:Lys15 C:Leu17
P-**3**	-5.75 / -4.58	3% (-5.75)	B:Ala109 B:Leu110 B:Ser117 B:Thr119 B:Lys15 B:Leu17 D:Ala108 D:Leu110 D:Thr119 D:Lys15 D:Leu17
		6% (-5.18)	A:Ala108 A:Leu110 A:Ser117 A:Thr119 A:Lys15 A:Leu17 C:Ala108 C:Ala109 C:Leu110 C:Thr119 C:Lys15 C:Leu17
M-**4**	-5.34 / -4.47	5% (-5.15)	B:Ala109 B:Leu110 B:Thr119 B:Lys15 B:Leu17 D:Ala108 D:Ala109 D:Thr119 D:Lys15 D:Leu17
P-**4**	-4.89 / -4.84	0	--
M-**5**	-5.65 / -4.45	0	--
P-**5**	-5.10 / -4.42	1% (-4.93)	B:Ala109 B:Leu110 B:Thr119 B:Val121 B:Lys15 B:Leu17 D:Ala108 D:Ala109 D:Thr119 D:Lys15 D:Leu17
**6**	-5.50 / -5.51	3% (-5.50)	B:Ala109 B:Leu110 B:Ser117 B:Thr119 B:Lys15 B:Leu17 D:Ala108 D:Leu110 D:Thr119 D:Lys15 D:Leu17
		4% (-5.47)	B:Leu110 B:Thr119 B:Leu17 D:Ala108 D:Leu110 D:Ser117 D:Thr119 D:Lys15 D:Leu17

[a] AutoDock 4.2.6 was employed as docking program, and the software Chimera 1.13.1 for the graphical representation of the poses derived from the docking calculation. [b] TTR structure released from crystal structure, PDB ID:1ICT (A. Wojtczak, P. Neumann, V. Cody, Acta Cryst. D57 (2001) 957-967).

Table S9
Docking results for 4,4'-bipyridines **1**-**6** in the T_4 binding pocket.

Bipy	E.F.E.B.[a]	E.I.C., K_i[b]	Interactions with amino acids	distance [Å]	
				HB, D: Lys15'	XB, D: Ser117'
(*M*)-**1**	-4.59	430.30	B: Ala109, Leu110, Lys15, Leu17<br>D: Thr106, Ala108, Thr119, Lys15, Leu17	1.9	
(*P*)-**1**	-4.01	1000.16	B: Ala109, Leu110, Thr119, Val121, Lys15, Leu17<br>D: Ala108, Thr119, Lys15, Leu17	2.1	
(*M*)-**2**	-5.72	64.06	B: Leu110, Thr119, Lys15, Leu17<br>D: Ala108, Ala109, Leu110, Ser117, Thr119, Leu17	--	
(*P*)-**2**	-5.62	75.81	B: Ala109, Leu110, Thr119, Lys15, Leu17<br>D: Ala108, Ala109, Leu110, Ser117, Thr119, Lys15, Leu17	--	
(*M*)-**3**	-6.56	15.65	B: Leu110, Thr119, Val121, Leu17<br>D: Ala108, Leu110, Ser117, Thr119, Lys15, Leu17	2.6	3.4 (I···O)
(*P*)-**3**	-5.96	42.69	B: Ala109, Leu110, Ser117, Thr119, Lys15, Leu17<br>D: Ala108, Leu110, Thr119, Lys15, Leu17	2.3	
(*M*)-**4**	-5.44	102.59	B: Ala109, Leu110, Thr119, Lys15, Leu17<br>D: Ala108, Ala109, Leu110, Thr119, Lys15, Leu17	--	
(*P*)-**4**	-5.26	138.27	B: Ala109, Leu110, Thr119, Lys15, Leu17<br>D: Ala108, Thr119, Lys15, Leu17	2.0	
(*M*)-**5**	-5.56	83.85	B: Ala109, Leu110, Thr119, Lys15, Leu17,<br>D: Ala108, Ala109, Leu110, Ser117, Thr119, Lys15, Leu17	--	
(*P*)-**5**	-5.36	118.52	B: Ala109, Leu110, Thr119, Val121, Lys15, Leu17<br>D: Ala108, Thr119, Lys15, Leu17	2.6	
**6**	-5.93	44.82	B: Leu110, Thr119, Leu17<br>D: Ala108, Leu110, Ser117, Thr119, Lys15, Leu17	2.6	3.4 (I···O)

[a] E.F.E.B. Estimated Free Energy of Binding [kcal/mol]. [b] E.I.C., K_i Estimated Inhibition Constant, K_i [μM].

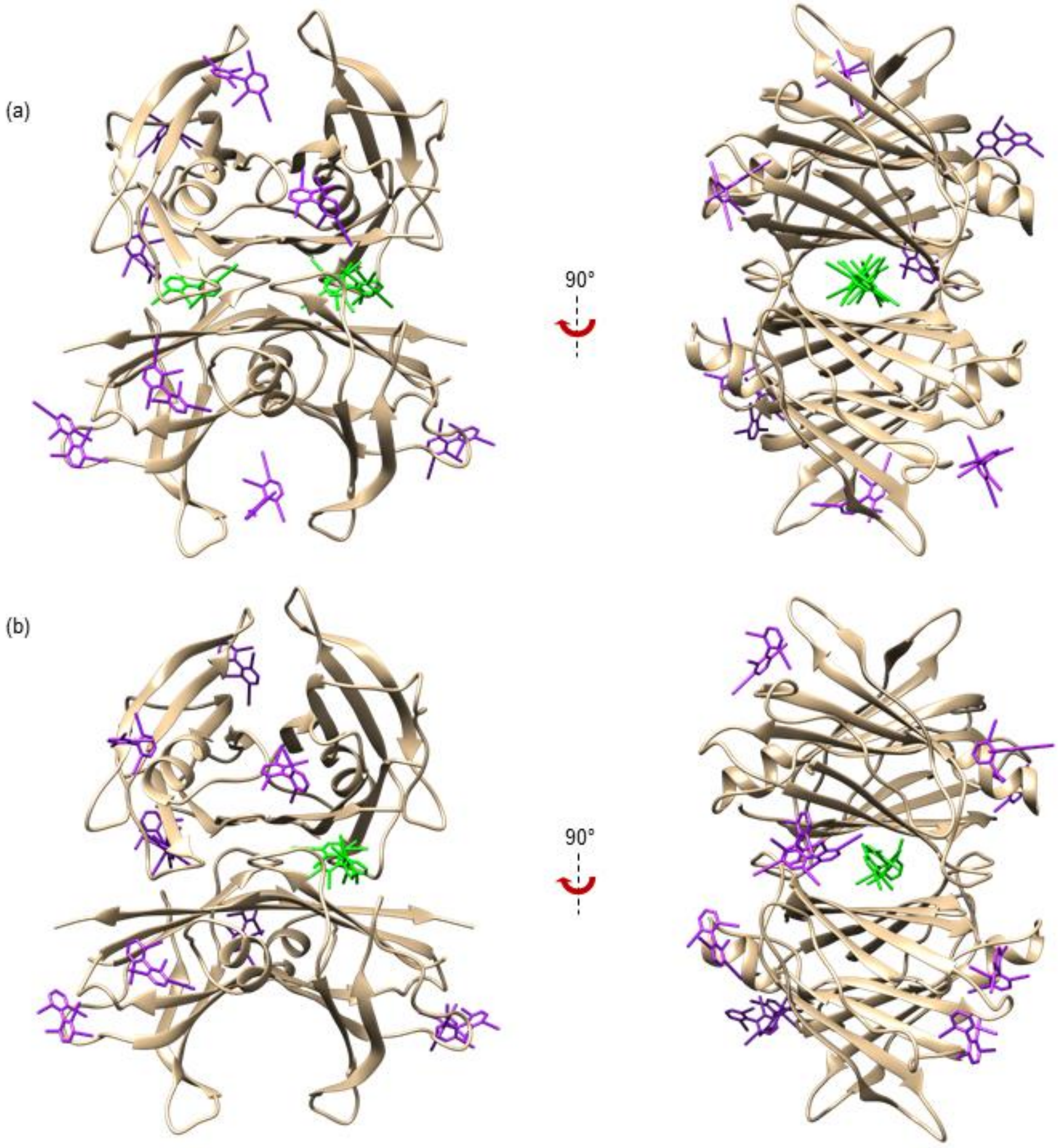

Figure S2. Blind docking poses of compounds (*M*)-**3** (a) and **6** (b) on the whole TTR (molecules found in the T_4 binding pockets are coloured in green).

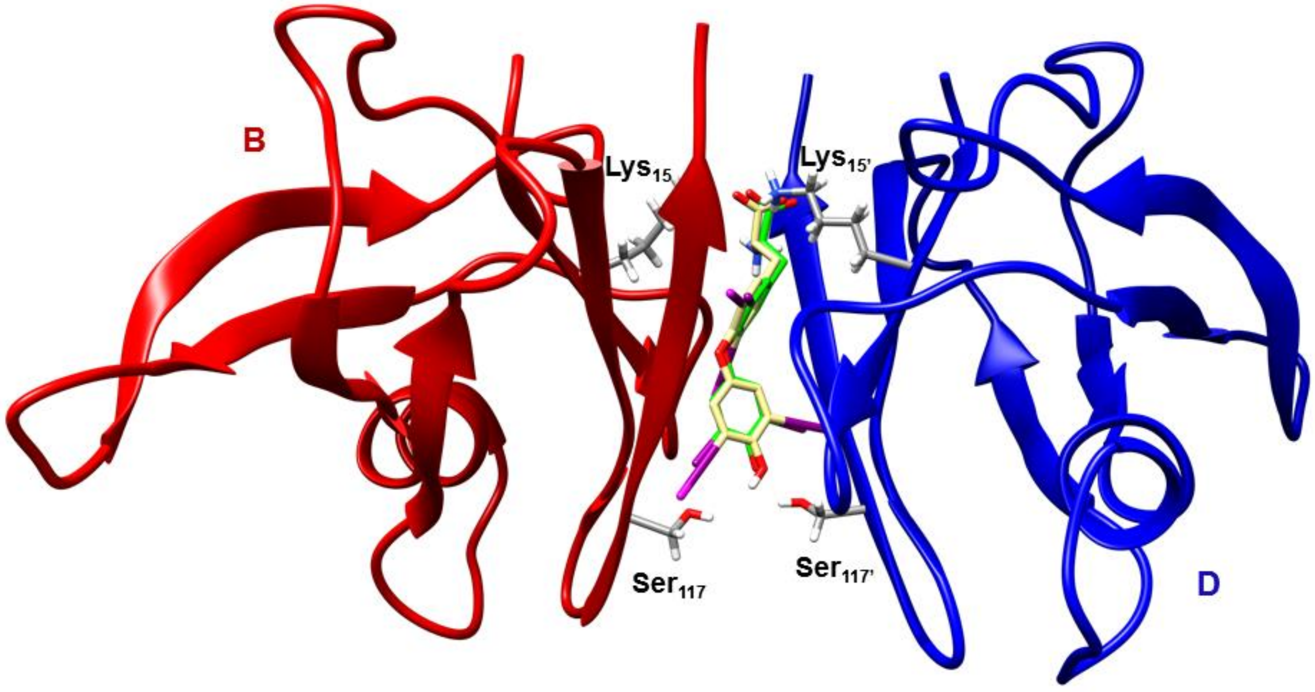

Figure S3. Comparison of the docked pose of T_4 (pale yellow) into TTR (BD:T_4 binding pocket) with the crystallographic structure of T_4 (released from PDB ID: 1ICT) (green).

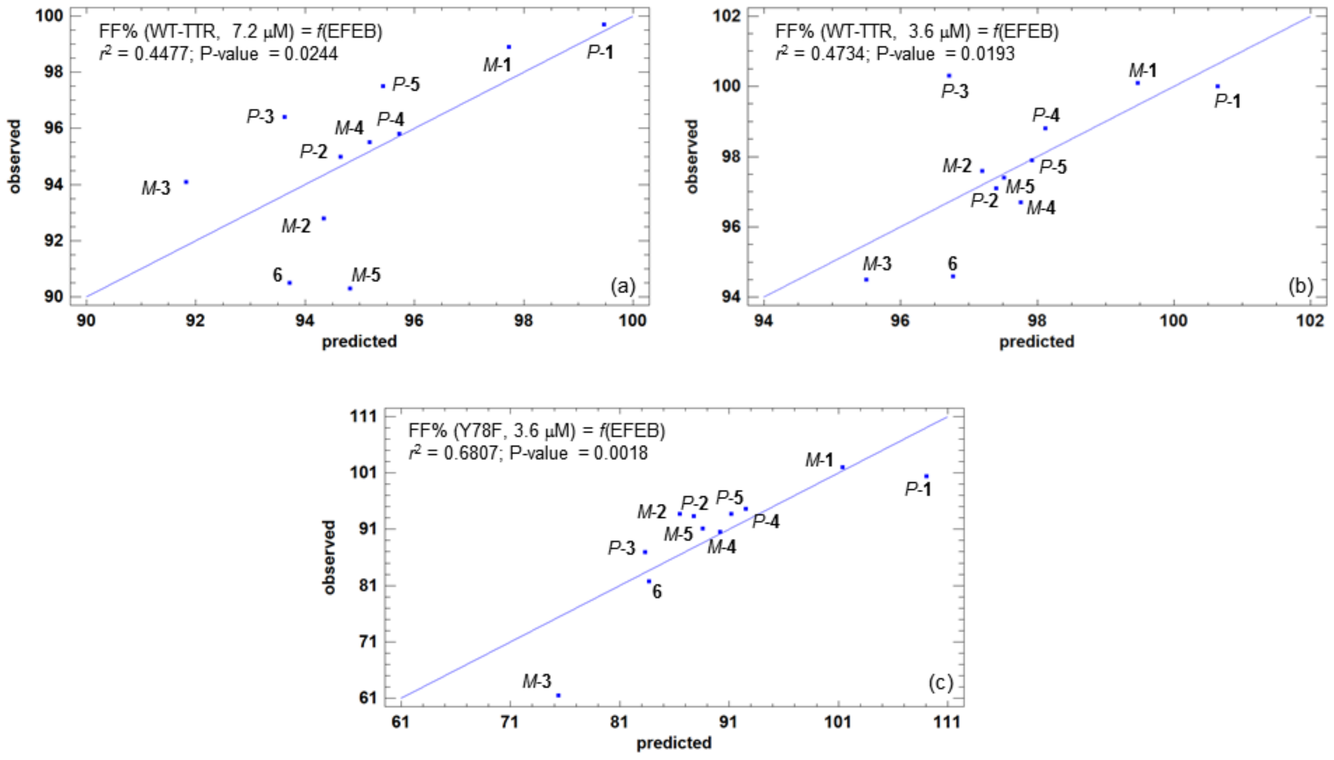

Figure S4. Linear regression analysis describing the relationships between FF% and calculated EFEB by docking in the T_4 pocket (BD): WT-TTR at 7.2 μM (a) and 3.6 μM (b) of inhibitor concentration, and (c) Y78F at 3.6 μM of inhibitor concentration.

S3. NMR spectra

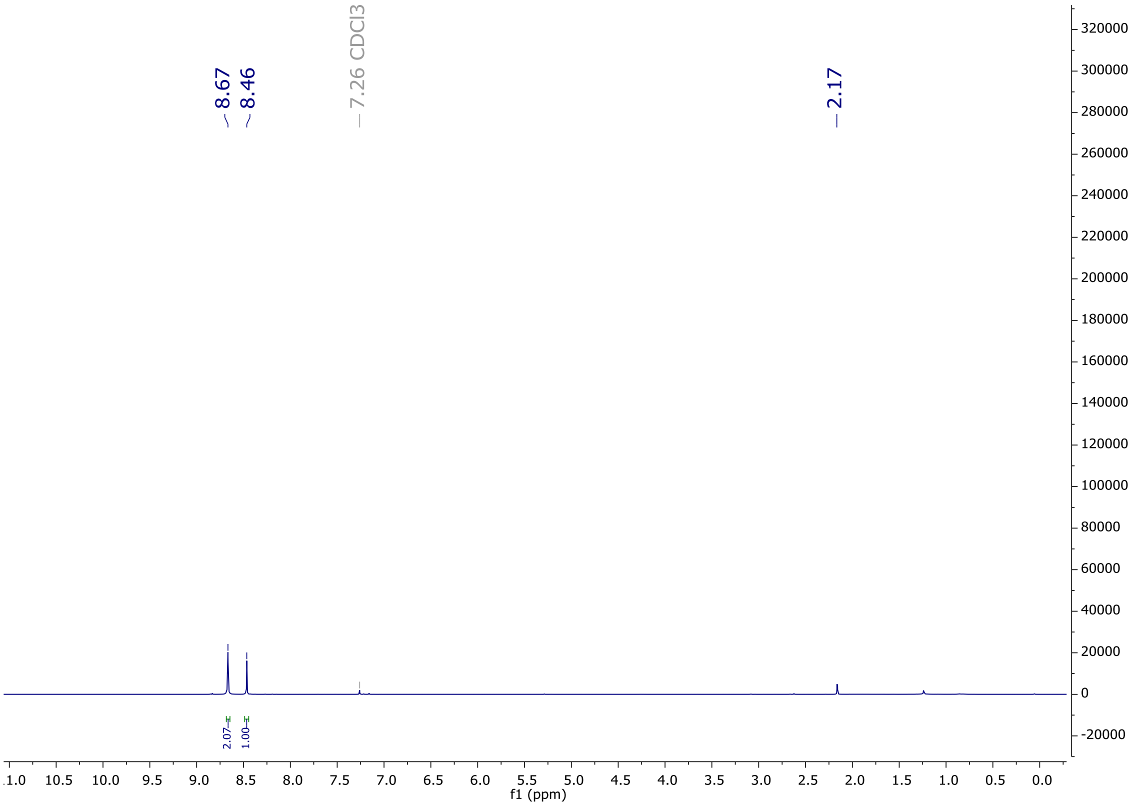

Figure S5. ^{1}H NMR spectrum of 3,3',5,5'-tetrachloro-2-iodo-4,4'-bipyridine (**6**).

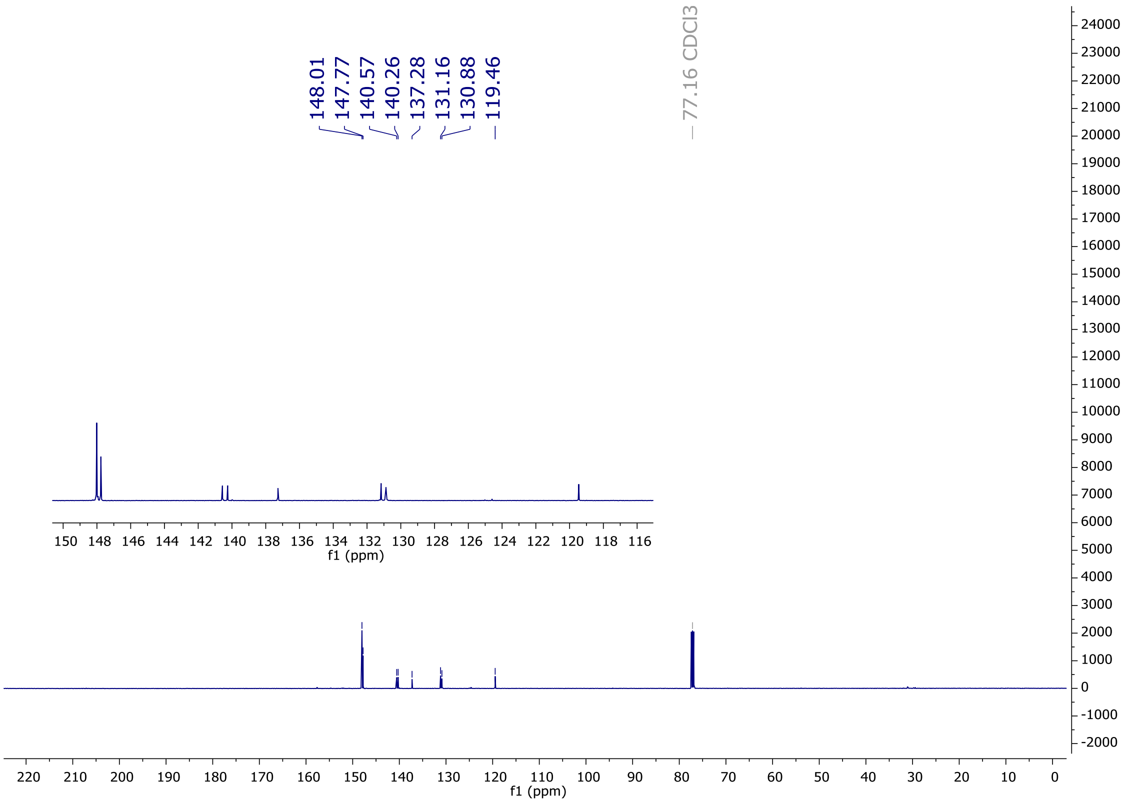

Figure S6. ^{13}C NMR spectrum of 3,3',5,5'-tetrachloro-2-iodo-4,4'-bipyridine (**6**).

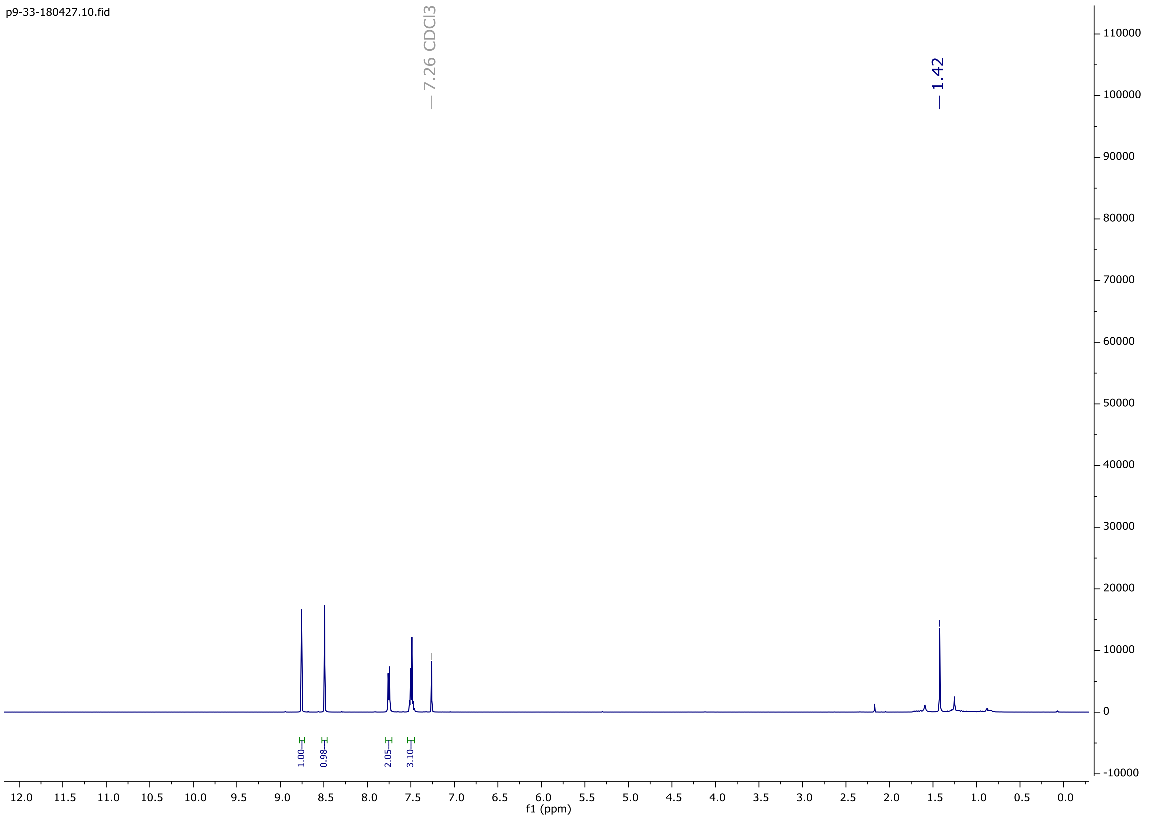

Figure S7. ^{1}H NMR spectrum of 3,3',5,5'-tetrachloro-2-iodo-2'-phenyl-4,4'-bipyridine (**7**).

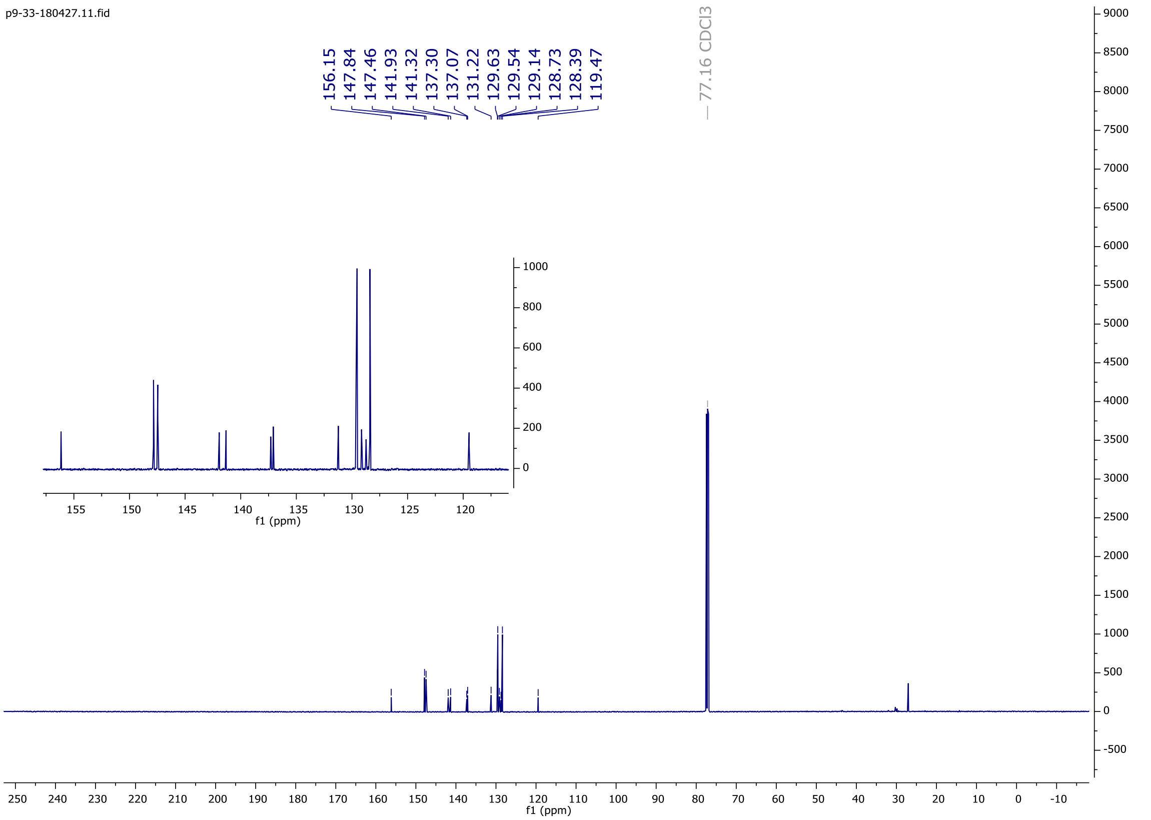

Figure S8. ^{13}C NMR spectrum of 3,3',5,5'-tetrachloro-2-iodo-2'-phenyl-4,4'-bipyridine (**7**).

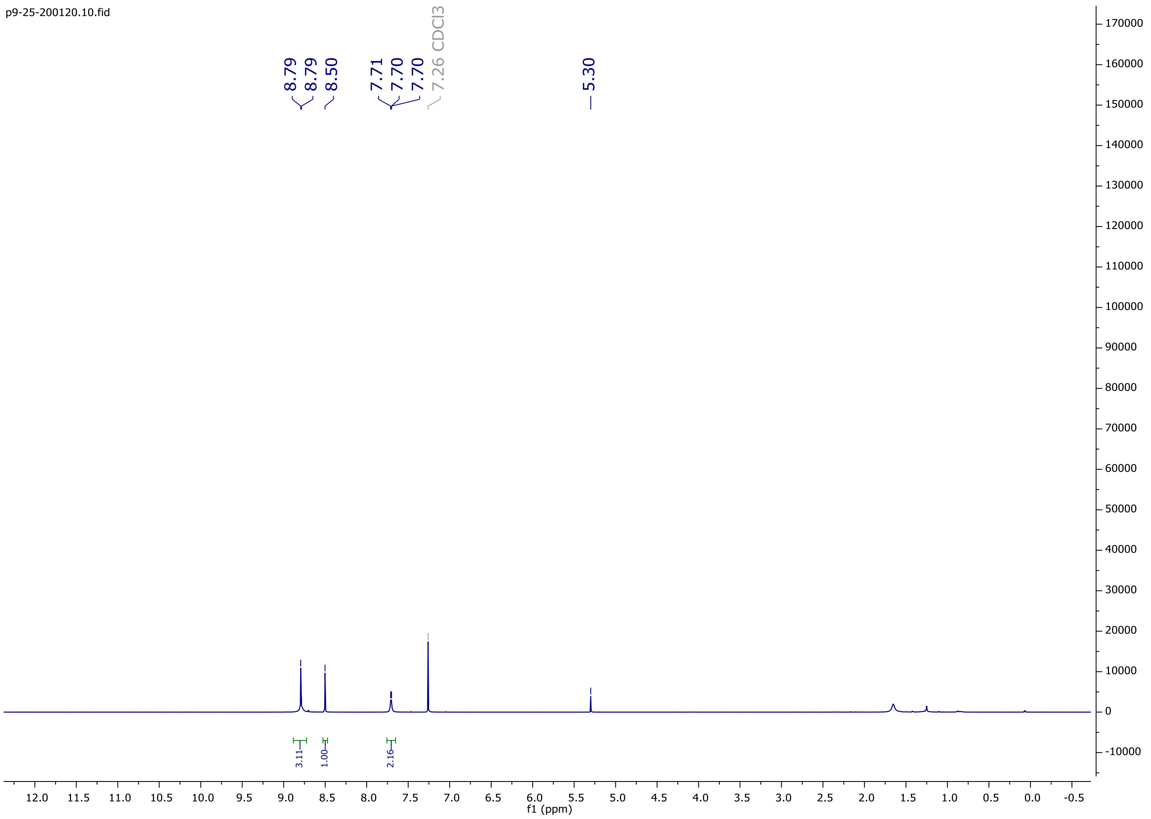

Figure S9. ^{1}H NMR spectrum of 3,3',5,5'-tetrachloro-2-iodo-2'-(4-pyridyl)-4,4'-bipyridine (**8**).

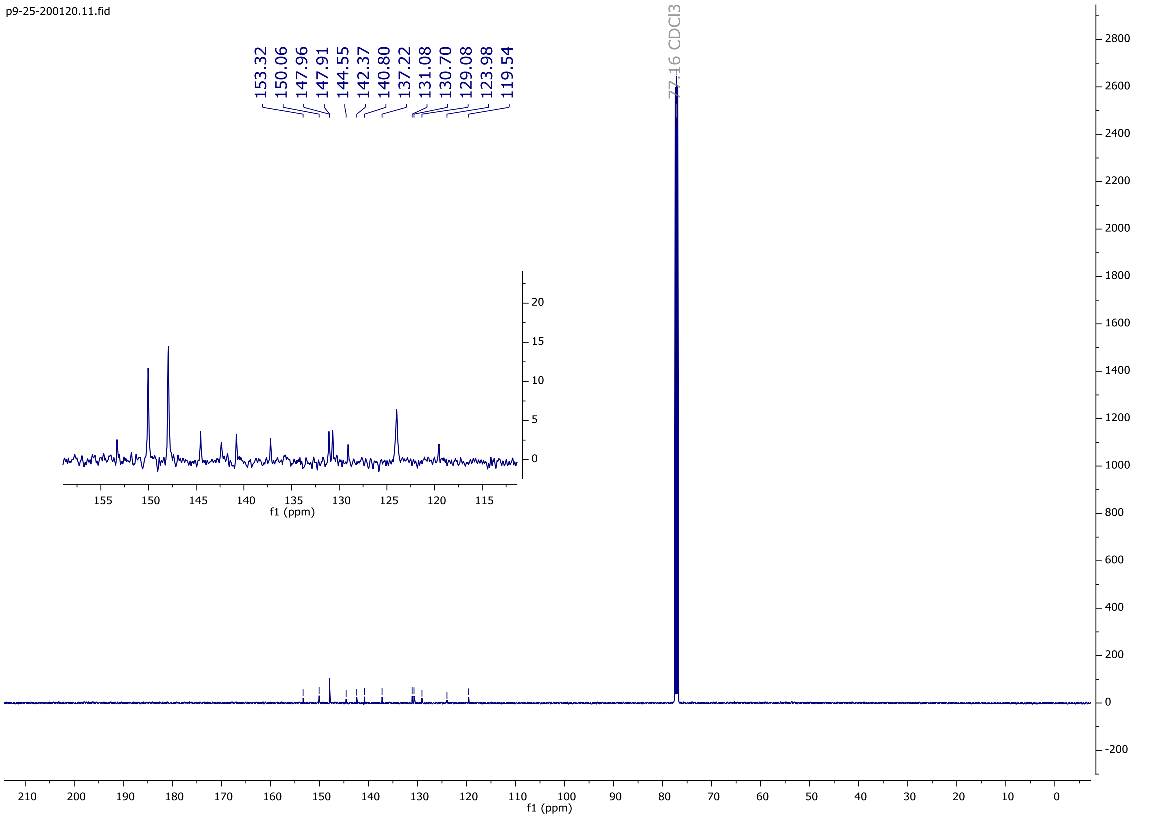

Figure S10. ^{13}C NMR spectrum of 3,3',5,5'-tetrachloro-2-iodo-2'-(4-pyridyl)-4,4'-bipyridine (**8**).

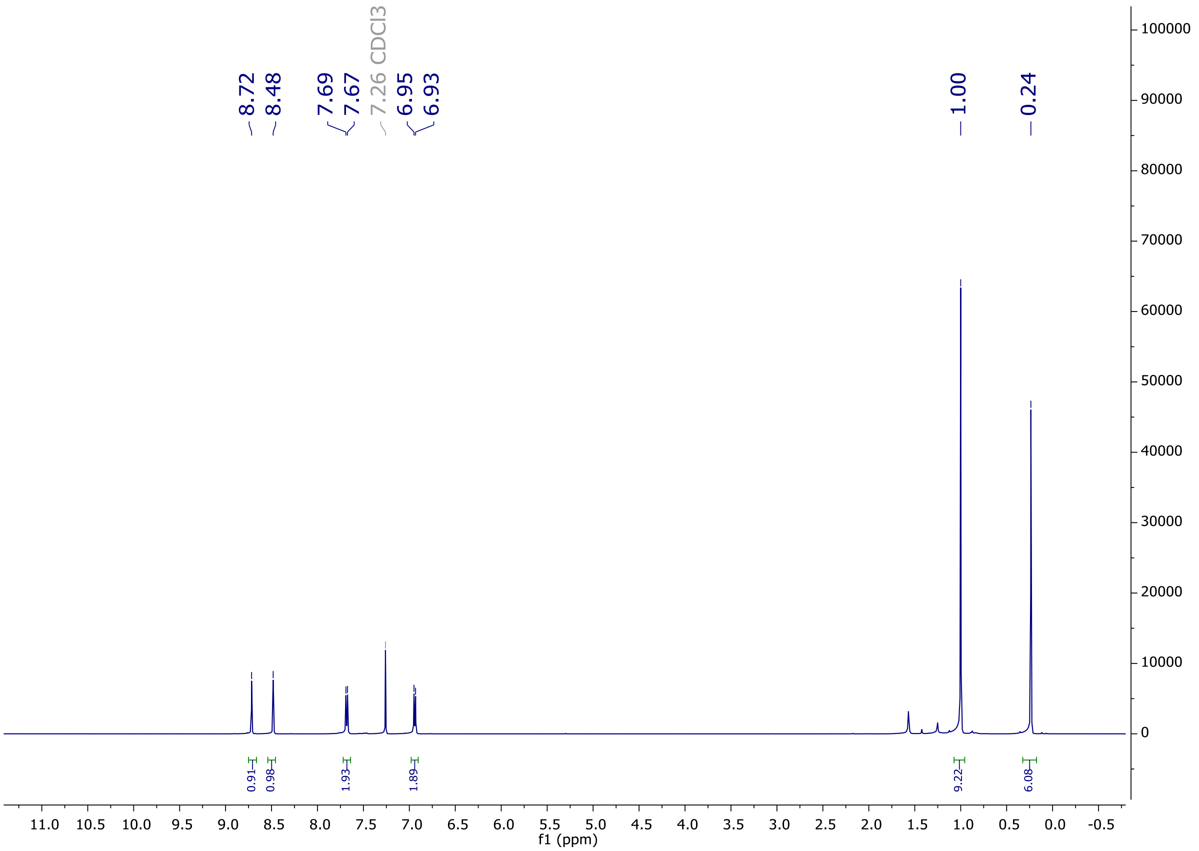

Figure S11. ^{1}H NMR spectrum of 3,3’,5,5’-tetrachloro-2-iodo-2’-(4-tert-butyldimethylsilyloxyphenyl)-4,4'-bipyridine (**14**).

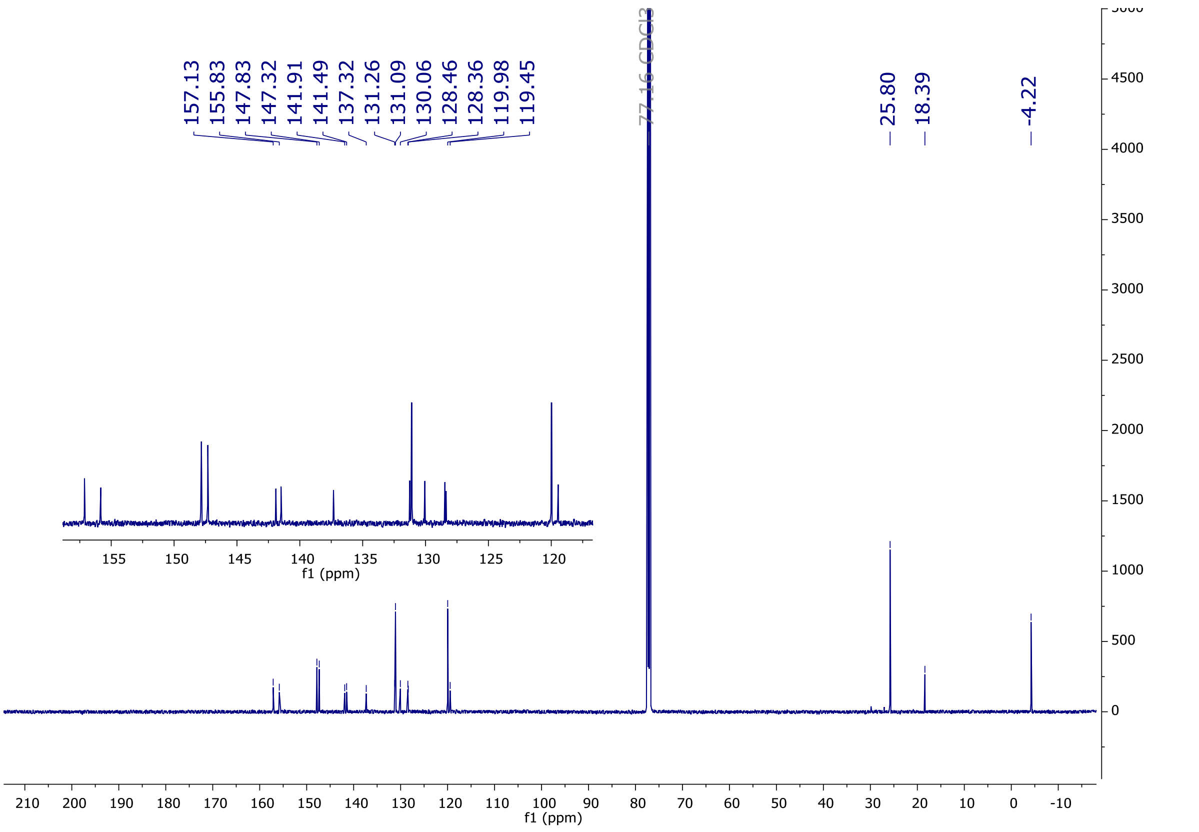

Figure S12. ^{13}C NMR spectrum of 3,3’,5,5’-tetrachloro-2-iodo-2’-(4-tert-butyldimethylsilyloxyphenyl)-4,4'-bipyridine (**14**).

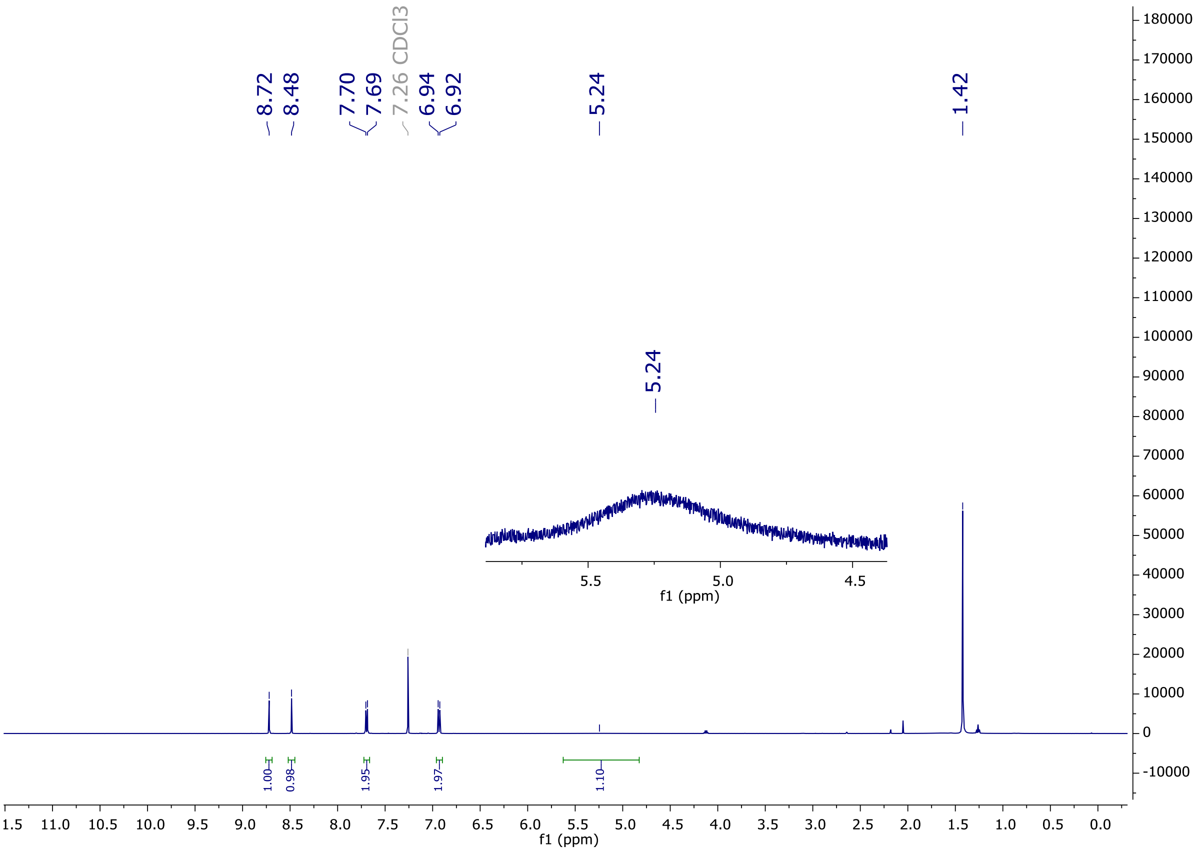

Figure S13. ^{1}H NMR spectrum of 3,3’,5,5’-tetrachloro-2-iodo-2’-(4-hydroxyphenyl)-4,4’-bipyridine (**9**).

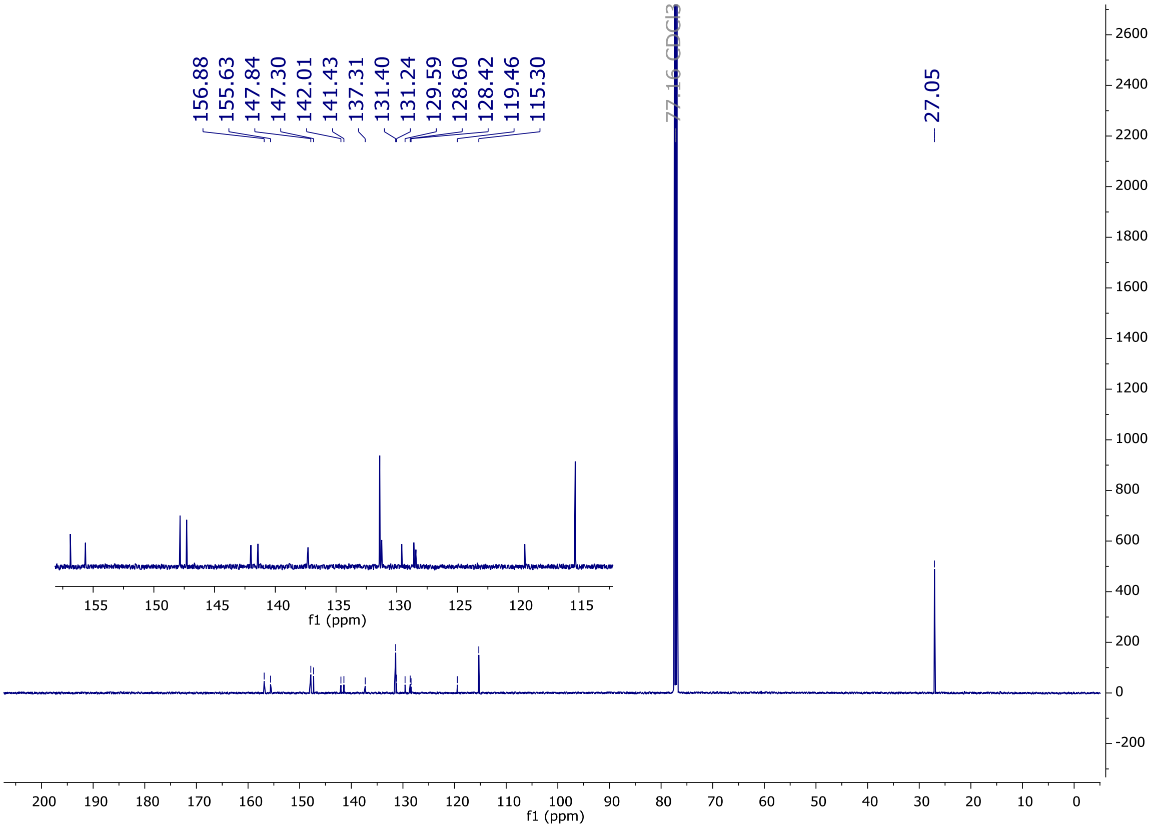

Figure S14. ^{13}C NMR spectrum of 3,3’,5,5’-tetrachloro-2-iodo-2’-(4-hydroxyphenyl)-4,4’-bipyridine (**9**).

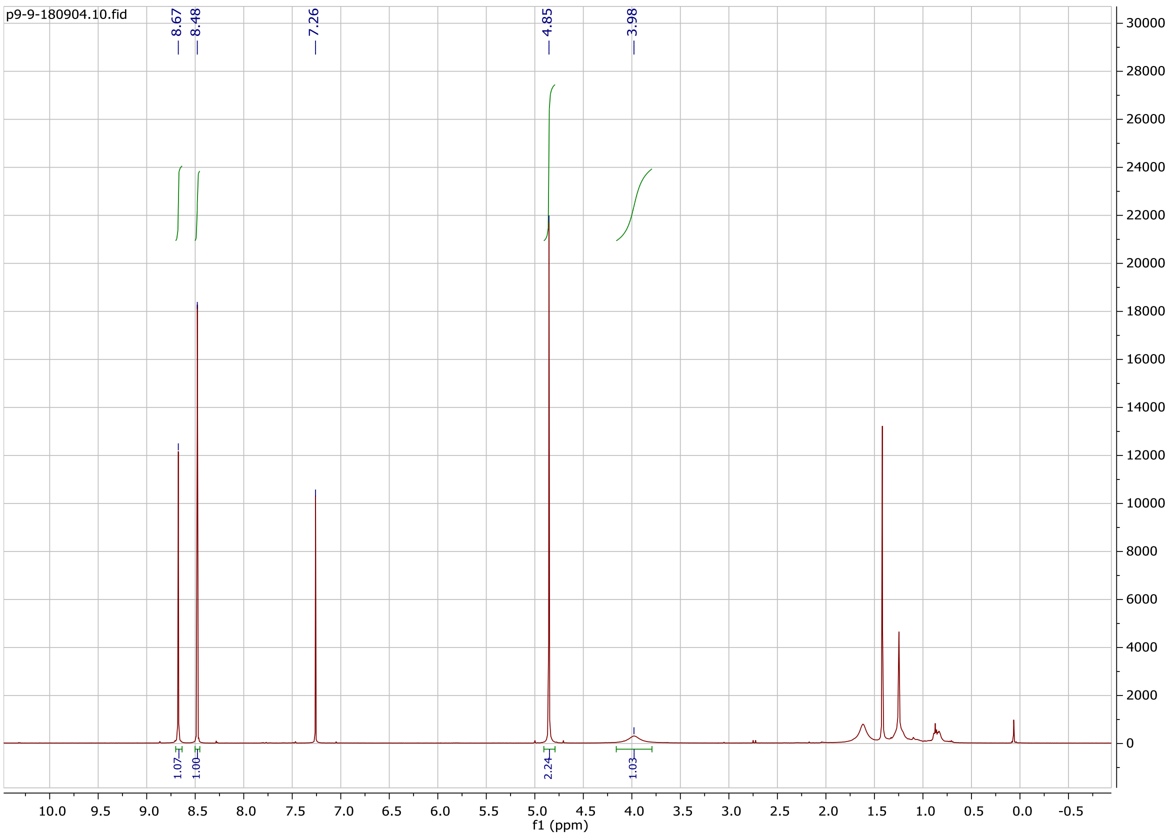

Figure S15. ^{1}H NMR spectrum of 3,3’,5,5’-tetrachloro-2-iodo-2’-hydroxymethyl-4,4’-bipyridine (**10**).

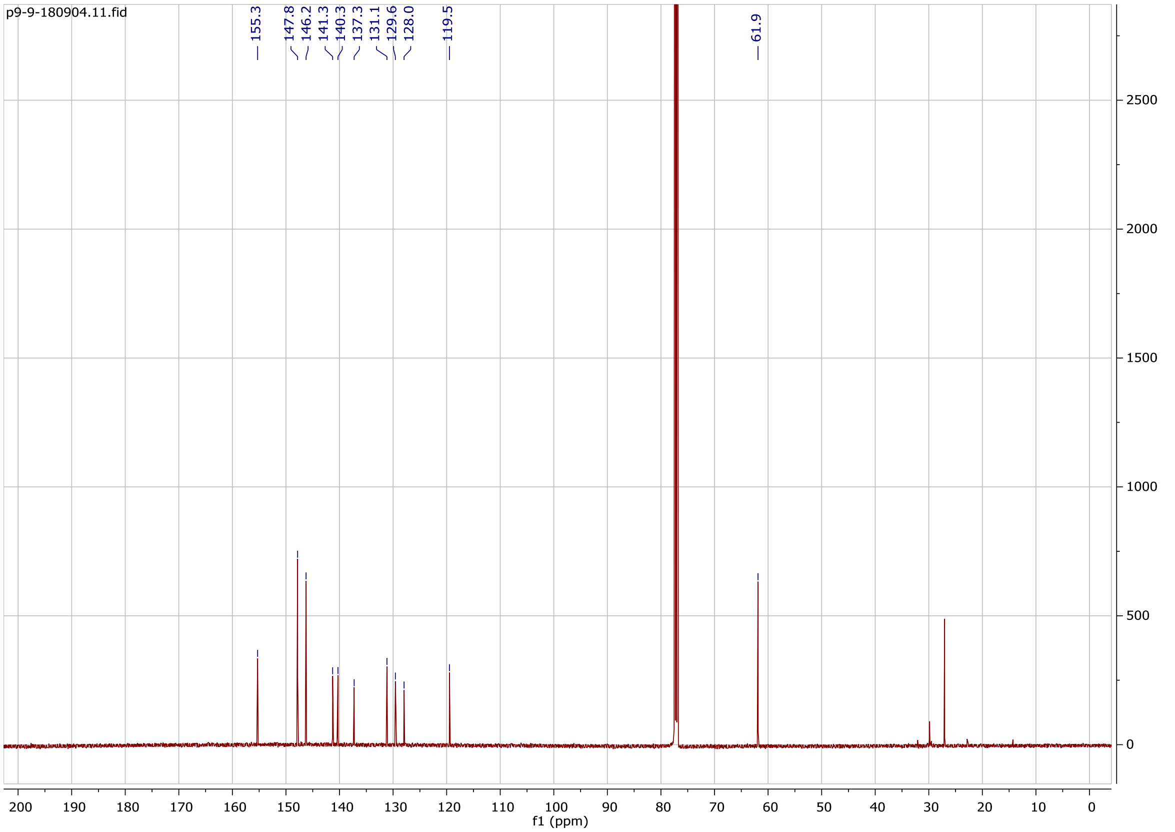

Figure S16. ^{13}C NMR spectrum of 3,3’,5,5’-tetrachloro-2-iodo-2’-hydroxymethyl-4,4’-bipyridine (**10**).

S4. HPLC enantioseparation

Table S10

Optimized multimilligram enantioseparation of 4,4'-bipyridines **7-10** [1].

Bipy	Racemate (mg)	absolute configuration (ee%[2])		recovered amounts (mg) (recovery%)	
		1st eluted peak (pk)	2nd eluted peak	1st eluted peak	2nd eluted pk
**7**	30.0	*P* (>99)	*M* (>95.7)	14.0 (93.3)	14.9 (99.3)
**8**	13.3	*P* (>99)	*M* (>99)	5.9 (88.7)	6.6 (99.2)
**9**	18.0	*P* (>99)	*M* (>99)	7.4 (82.2)	8.7 (96.7)
**10**	26.0	*P* (>99)	*M* (>95.6)	12.2 (93.8)	11.1 (85.4)

[1] Mobile phase: *n*-hexane/2-propanol 90:10 (**7** and **10**), *n*-hexane/2-propanol/methanol 90:5:5 (**8** and **9**), T = 22°C. Flow rate (*FR*): 0.5 ml/min (**7**) and 0.8 ml/min (**8**-**10**).

[2] Enantiomeric excess (ee) determined by chiral HPLC under the same conditions used for recoveries.

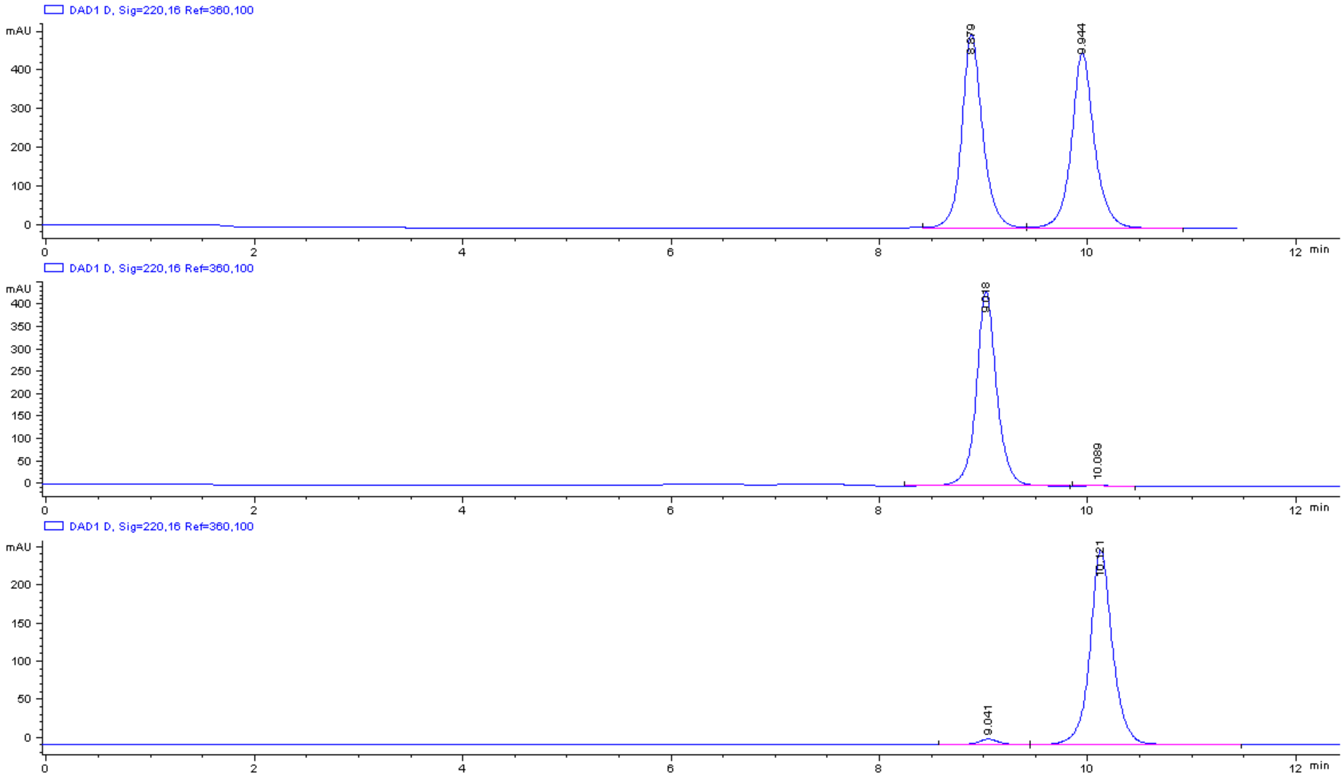

(*P*)-**7**

Signal 2: DAD1 D, Sig=220,16 Ref=360,100

Peak #	RetTime [min]	Type	Width [min]	Area [mAU*s]	Height [mAU]	Area%
1	9.018	BB	0.2446	8701.84766	421.66461	100.0000

(*M*)-**7**

Signal 2: DAD1 D, Sig=220,16 Ref=360,100

Peak #	RetTime [min]	Type	Width [min]	Area [mAU*s]	Height [mAU]	Area%
1	9.041	BB	0.2179	110.37134	1.33176	2.1778
2	10.121	BB	0.2763	5068.02100	270.86273	97.8835

Figure S17. Enantioseparation of *rac*-**7** on Chiralpak IA, *n*-hexane/2-propanol 90:10, *FR* 0.5 ml/min, 220 nm.

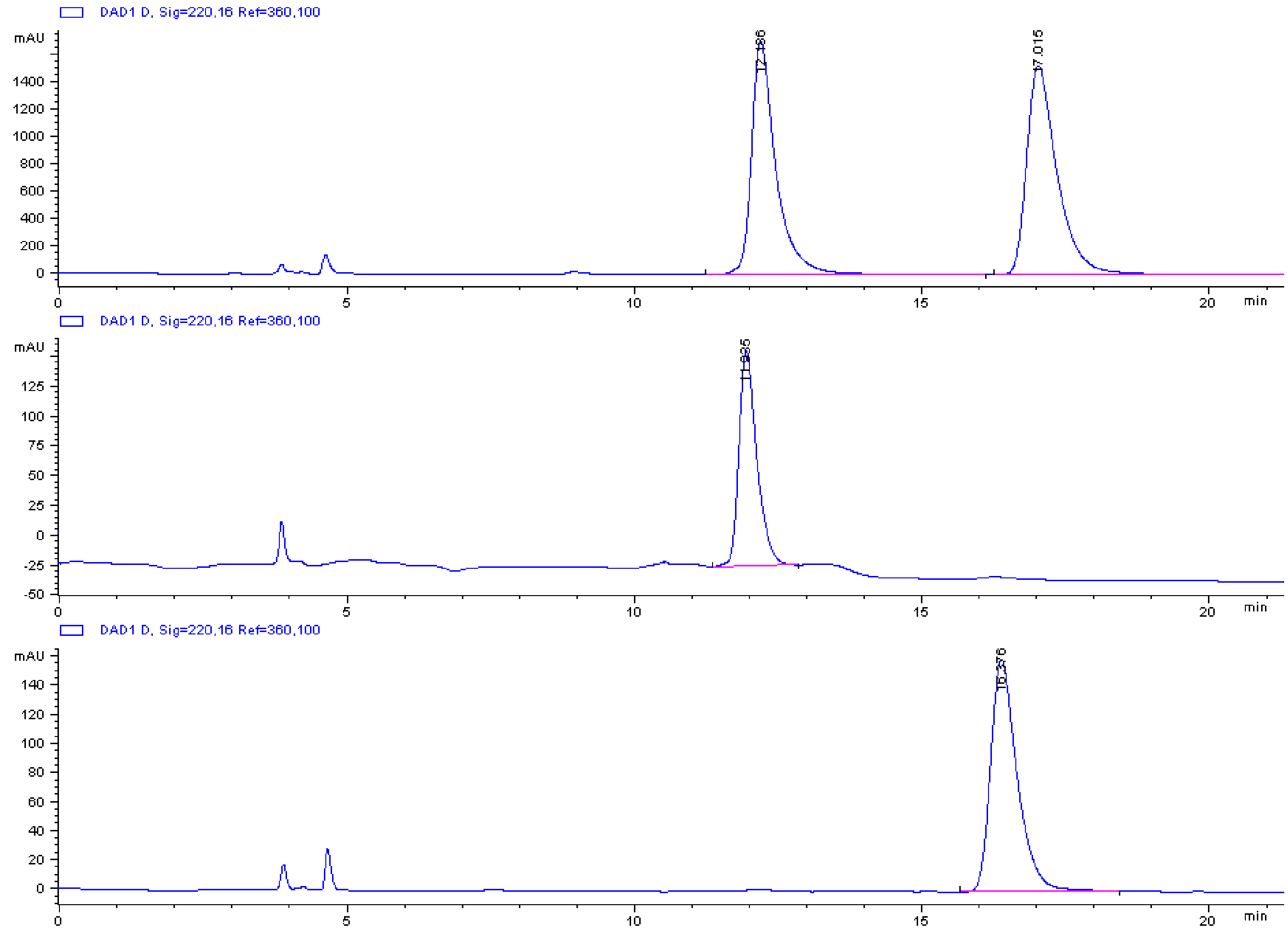

(*P*)-**8**

Signal 2: DAD1 D, Sig=220,16 Ref=360,100

Peak #	RetTime [min]	Type	Width [min]	Area [mAU*s]	Height [mAU]	Area%
1	11.935	BB	0.3232	3927.70581	180.99869	100.0000

(*M*)-**8**

Signal 2: DAD1 D, Sig=220,16 Ref=360,100

Peak #	RetTime [min]	Type	Width [min]	Area [mAU*s]	Height [mAU]	Area%
2	16.376	BB	0.4824	5122.97998	159.64221	100.0000

Figure S18. Enantioseparation of *rac*-**8** on Chiralcel OD-H, *n*-hexane/2-propanol/MeOH 90:5:5, *FR* 0.8 ml/min, 220 nm.

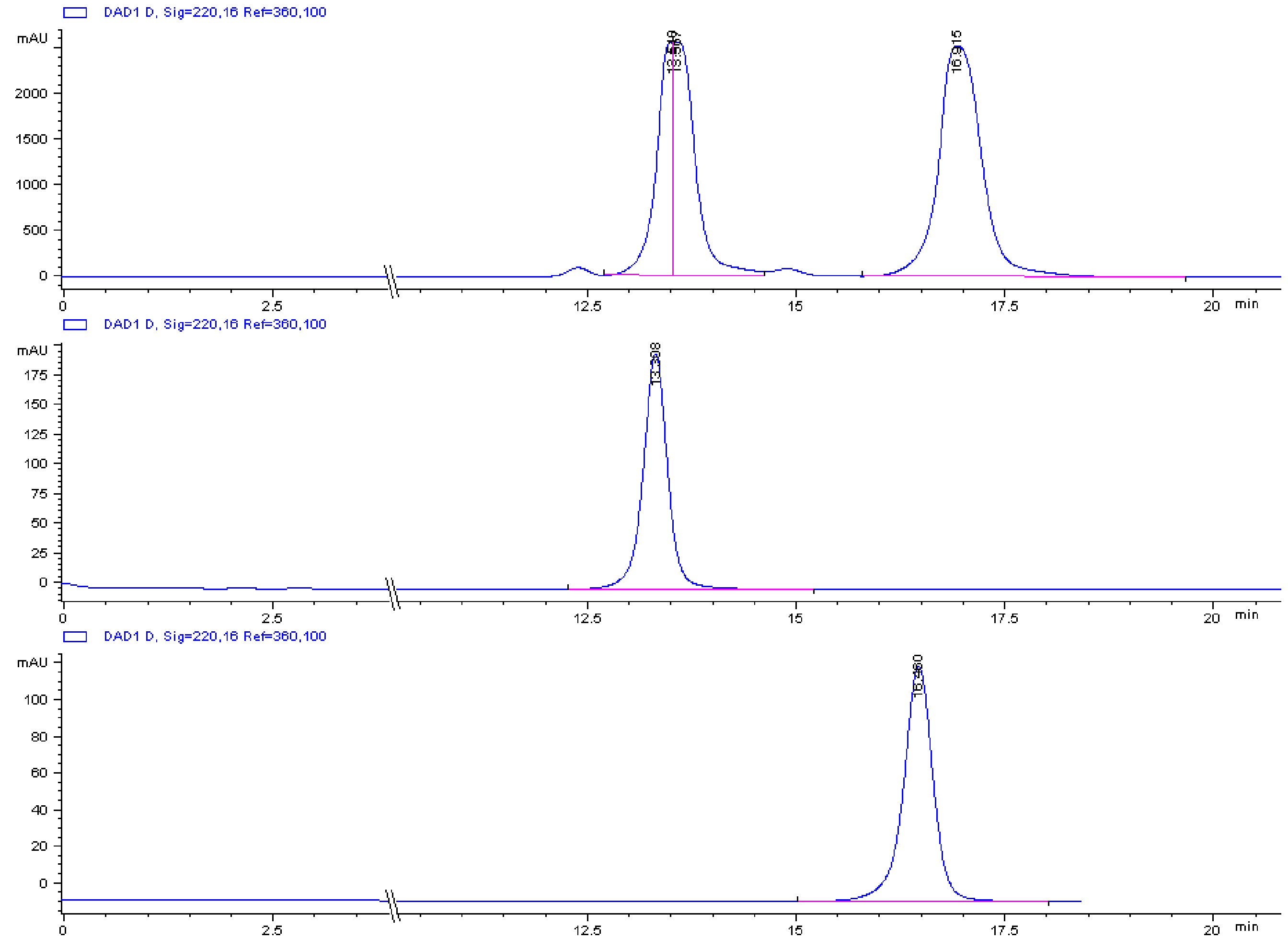

(*P*)-**9**

Signal 2: DAD1 D, Sig=220,16 Ref=360,100

Peak #	RetTime [min]	Type	Width [min]	Area [mAU*s]	Height [mAU]	Area%
1	13.308	BB	0.3114	4108.83350	198.56049	100.0000

(*M*)-**9**

Signal 2: DAD1 D, Sig=220,16 Ref=360,100

Peak #	RetTime [min]	Type	Width [min]	Area [mAU*s]	Height [mAU]	Area%
2	16.460	BB	0.3732	3207.65356	128.52205	100.0000

Figure S19. Enantioseparation of *rac*-**9** on Chiralpak IA, *n*-hexane/2-propanol/MeOH 90:5:5, *FR* 0.8 ml/min, 220 nm.

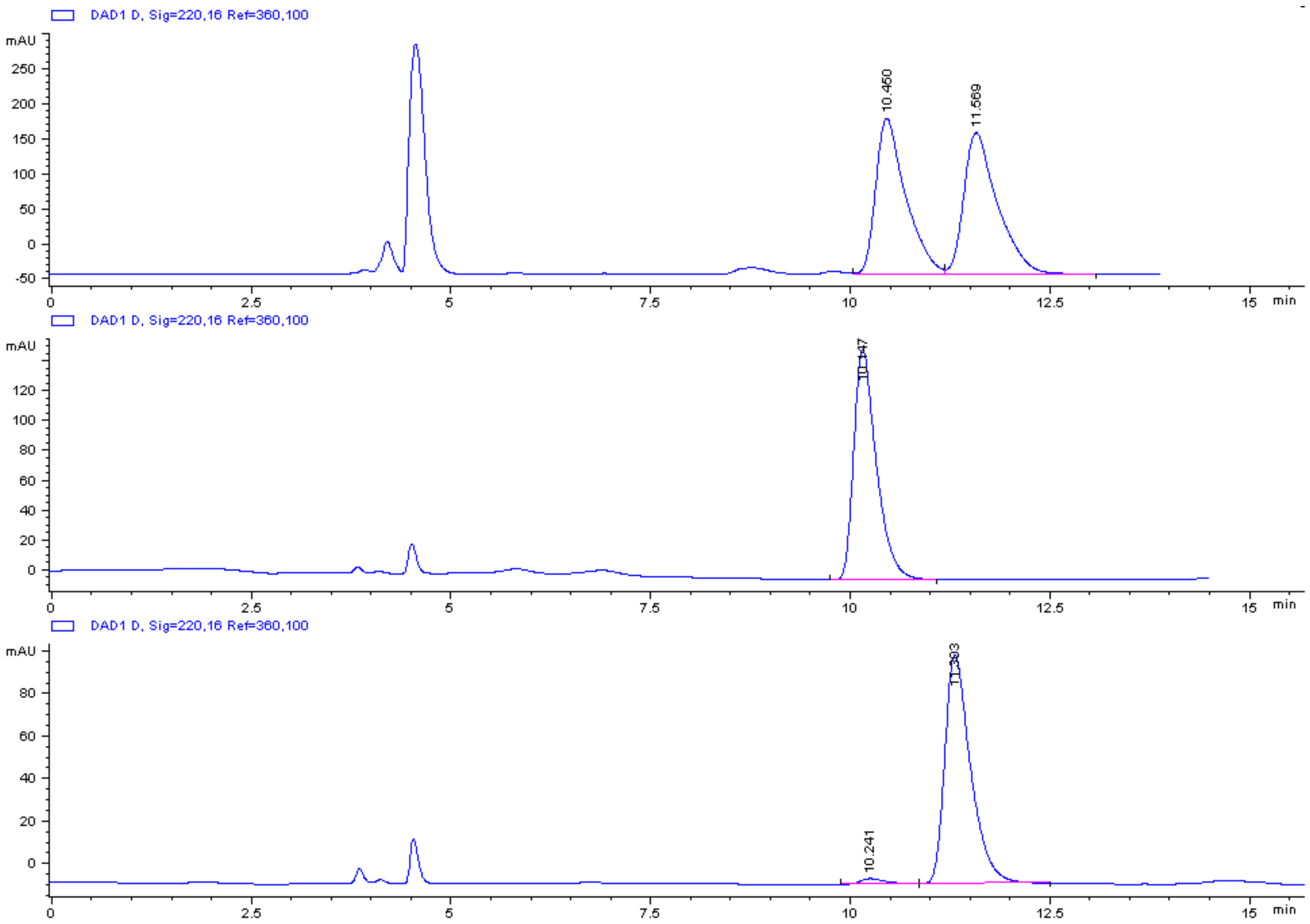

(*P*)-**10**

Signal 2: DAD1 D, Sig=220,16 Ref=360,100

Peak #	RetTime [min]	Type	Width [min]	Area [mAU*s]	Height [mAU]	Area%
1	10.147	BB	0.2952	3034.07813	154.44859	100.0000

(*M*)-**10**

Signal 2: DAD1 D, Sig=220,16 Ref=360,100

Peak #	RetTime [min]	Type	Width [min]	Area [mAU*s]	Height [mAU]	Area%
1	10.241	BB	0.3126	54.99158	2.60178	2.2158
2	11.303	BB	0.3366	2426.77661	107.82089	97.7842

Figure S20. Enantioseparation of *rac*-**10** on Chiralcel OD-H, *n*-hexane/2-propanol 90:10, *FR* 0.8 ml/min, 220 nm

S5. Electronic circular dicroism (ECD) spectra of pure enantiomers of compounds 7-10

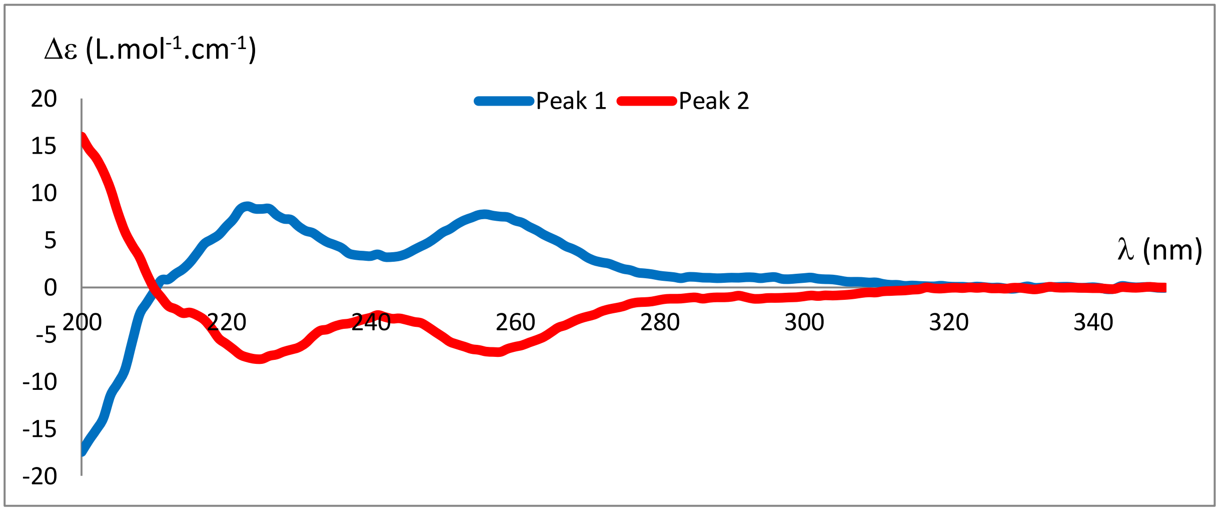

Figure S21. Experimental ECD spectra for the enantiomers of compound **7**.

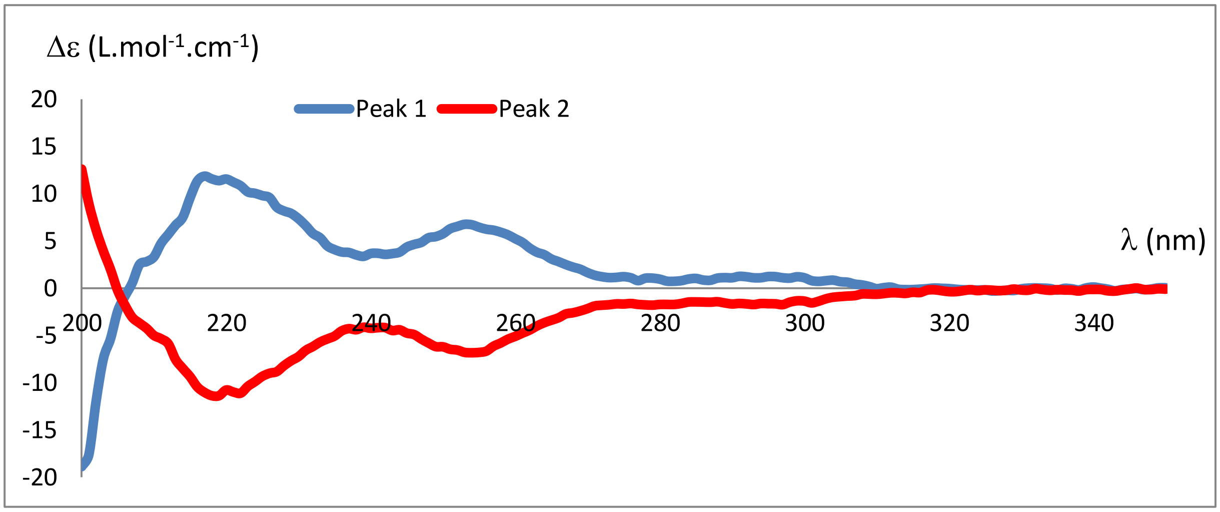

Figure S22. Experimental ECD spectra for the enantiomers of compound **8**.

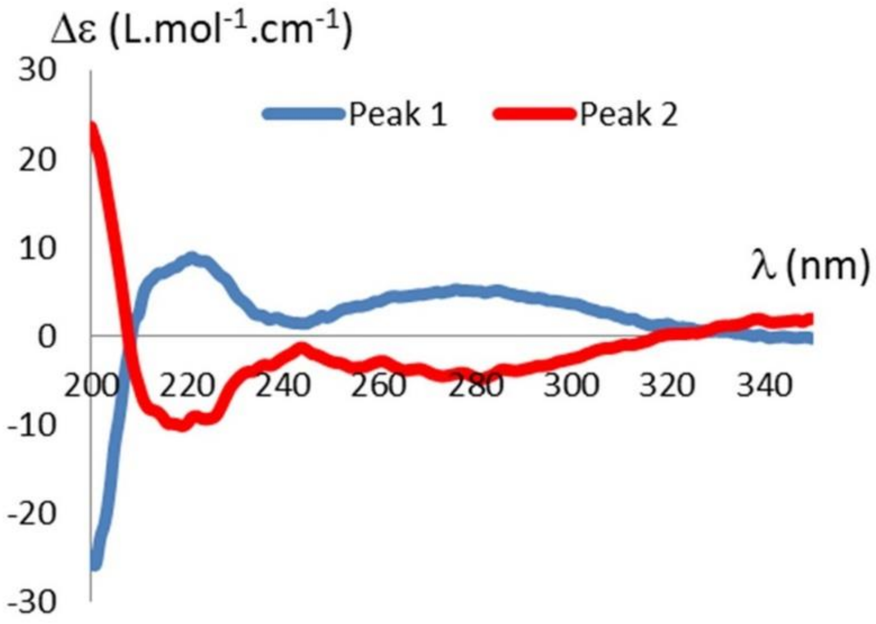

Figure S23. Experimental ECD spectra for the enantiomers of compound **9**.

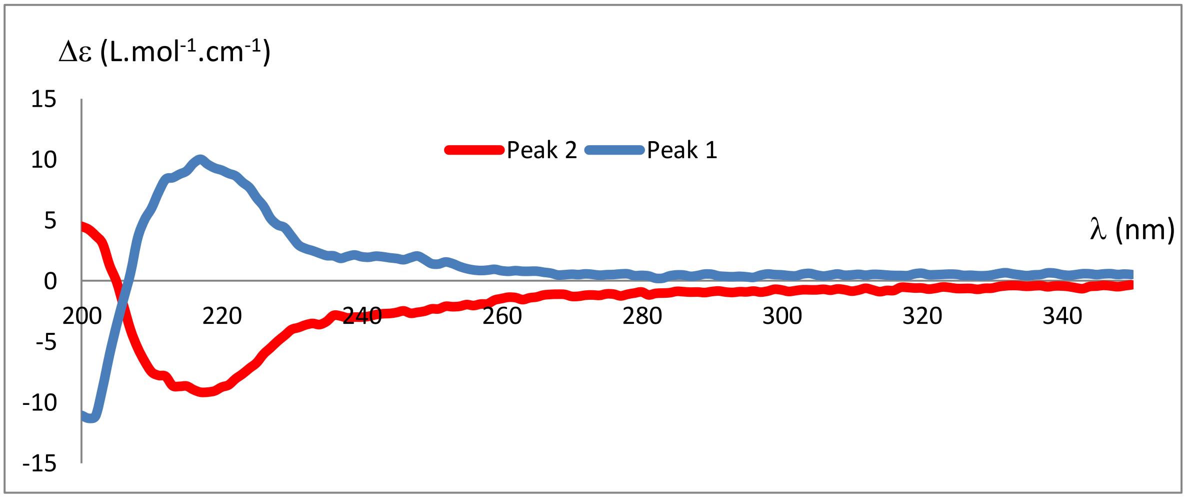

Figure S24. Experimental ECD spectra for the enantiomers of compound **10**.

S6. Absolute configuration assignment

Absolute configurations were obtained by comparison of experimental and theoretical electronic circular dichroism spectra [1]. Molecular conformations of bipys **7-10** were explored at the Density Functional Theory level of theory, using the CAM-B3LYP functional (completed with GD3 Grimme dispersion corrections [2]) and the aug-cc-pVTZ basis set for all atoms (including a pseudo-potential for Iodine) taken from EMSL library [3]. Solvent effects (ethanol) were taken into account through a PCM model. Frequency calculations, done at the same level of theory, proved that true energy minima were obtained for each conformer and Gibbs free energies were used to calculate populations based on Boltzmann statistics. ECD spectra were then obtained for each conformer by TD-DFT calculations (48 excited states, same functional, basis set & solvent modeling; half-width at half-height of 0.3 eV) and final spectra were simulated for each 4,4'-bipyridine by weighting the individual conformer spectra with the Boltzmann populations previously calculated.

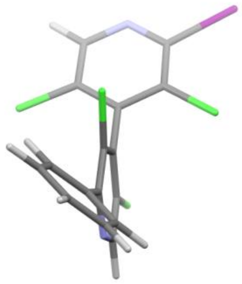
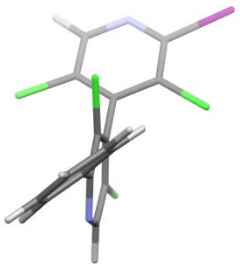

Compound **7**		
	Conf1	Conf2
G a.u.	-2859.798926	-2859.798929
ΔG (k/mol)	0.01	0.00
Population%	0.499	0.501

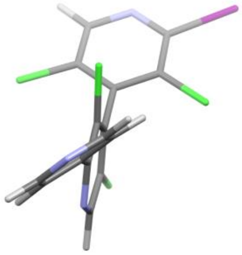
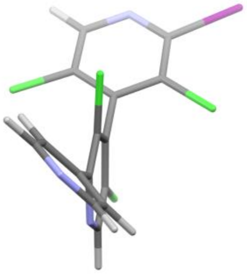

Compound **8**		
	Conf1	Conf2
G a.u.	-2875.853328	-2875.853323
ΔG (k/mol)	0.00	0.01
Population%	0.501	0.499

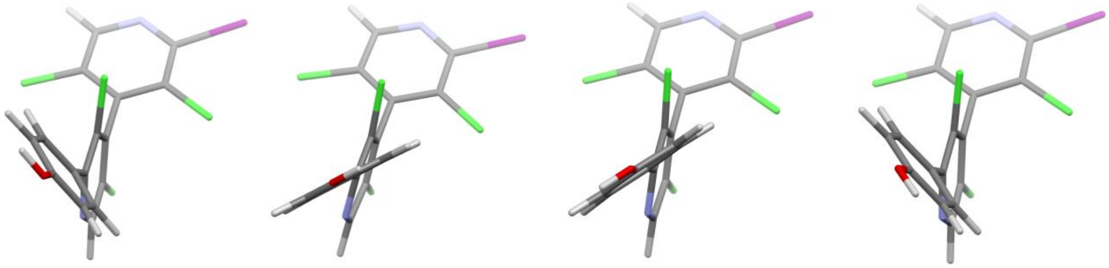

Compound **9**				
	Conf1	Conf2	Conf3	Conf4
G a.u.	-2935.033585	-2935.033576	-2935.033592	-2935.033611
ΔG (k/mol)	0.07	0.02	0.05	0.00
Population%	0.247	0.251	0.249	0.254

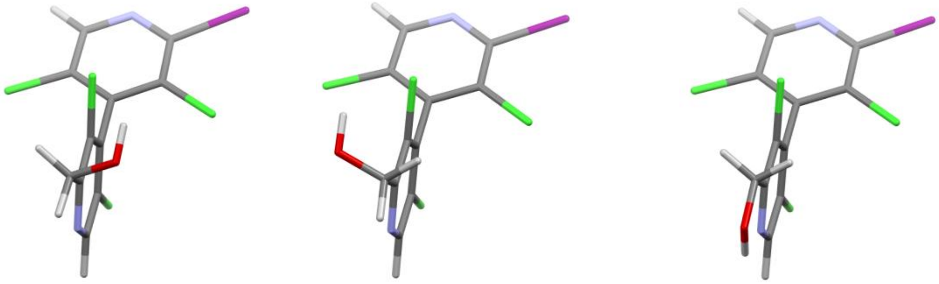

Compound **10**			
	Conf1	Conf2	Conf3
G a.u.	-2743.37346	-2743.37349	-2743.37845
ΔG (k/mol)	13.09	13.01	0.00
Population%	0.005	0.005	0.990

[1] Gaussian 09, Revision D.01, M. J. Frisch, G. W. Trucks, H. B. Schlegel, G. E. Scuseria, M. A. Robb, J. R. Cheeseman, G. Scalmani, V. Barone, B. Mennucci, G. A. Petersson, H. Nakatsuji, M. Caricato, X. Li, H. P. Hratchian, A. F. Izmaylov, J. Bloino, G. Zheng, J. L. Sonnenberg, M. Hada, M. Ehara, K. Toyota, R. Fukuda, J. Hasegawa, M. Ishida, T. Nakajima, Y. Honda, O. Kitao, H. Nakai, T. Vreven, J. A. Montgomery, Jr., J. E. Peralta, F. Ogliaro, M. Bearpark, J. J. Heyd, E. Brothers, K. N. Kudin, V. N. Staroverov, T. Keith, R. Kobayashi, J. Normand, K. Raghavachari, A. Rendell, J. C. Burant, S. S. Iyengar, J. Tomasi, M. Cossi, N. Rega, J. M. Millam, M. Klene, J. E. Knox, J. B. Cross, V. Bakken, C. Adamo, J. Jaramillo, R. Gomperts, R. E. Stratmann, O. Yazyev, A. J. Austin, R. Cammi, C. Pomelli, J. W. Ochterski, R. L. Martin, K. Morokuma, V. G. Zakrzewski, G. A. Voth, P. Salvador, J. J. Dannenberg, S. Dapprich, A. D. Daniels, O. Farkas, J. B. Foresman, J. V. Ortiz, J. Cioslowski, and D. J. Fox, Gaussian, Inc., Wallingford CT, 2013.

[2] S. Grimme, J. Antony, S. Ehrlich and H. Krieg, A consistent and accurate ab initio parameterization of density functional dispersion correction (DFT-D) for the 94 elements H-Pu, J. Chem. Phys. 132 (2010) 154104.

[3] B.P. Pritchard, D. Altarawy, B. Didier, T.D. Gibson, T.L. Windus, A new basis set exchange: an open, up-to-date resource for the molecular sciences community, J. Chem. Inf. Model. 59 (2019) 4814-4820.

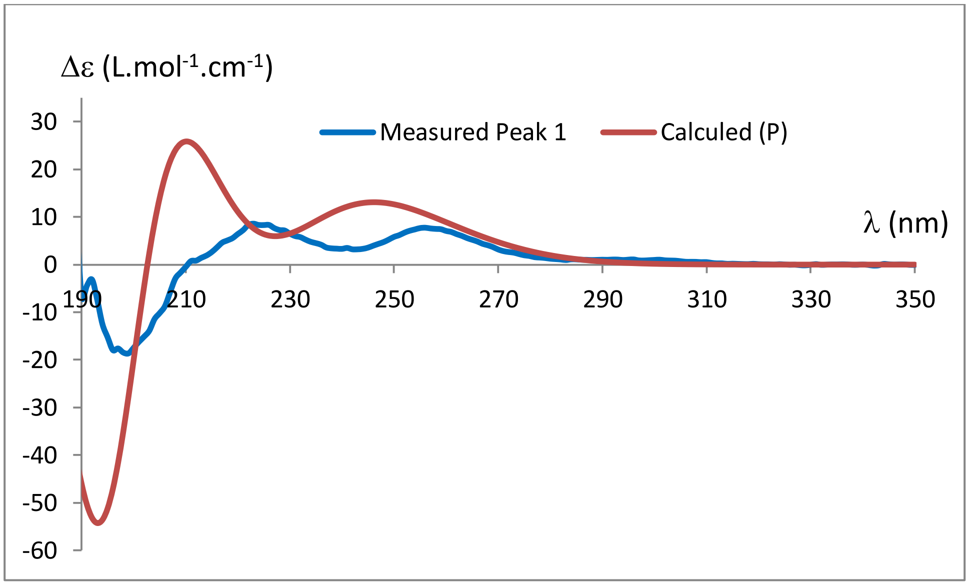

Figure S25. Comparison of measured and calculated ECD spectra for (*P*)-**7**.

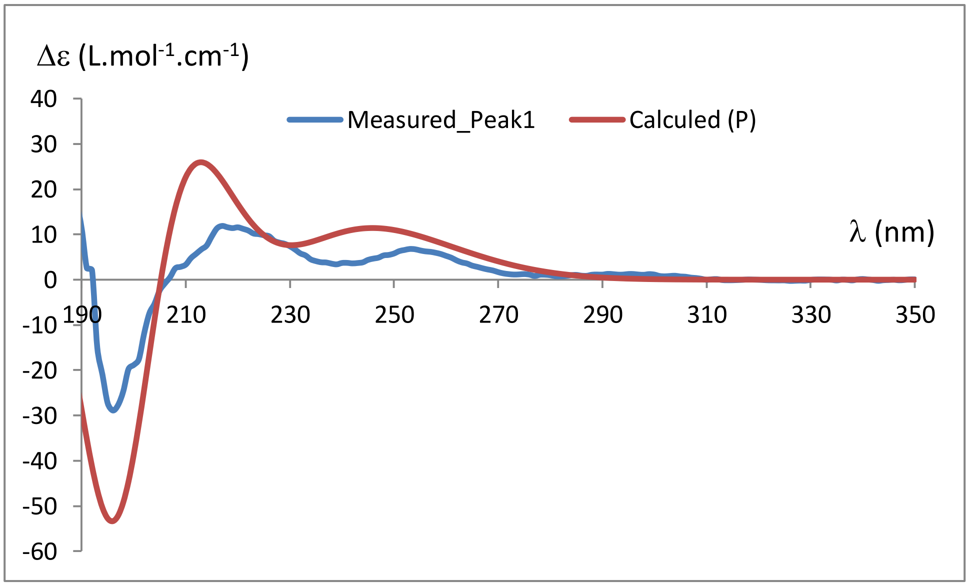

Figure S26. Comparison of measured and calculated ECD spectra for (*P*)-**8**.

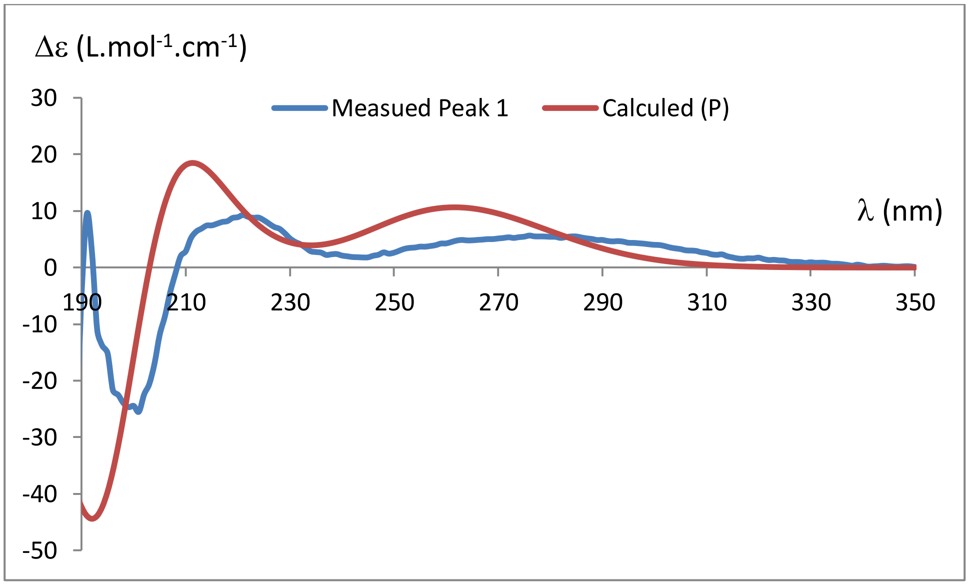

Figure S27. Comparison of measured and calculated ECD spectra for (*P*)-**9**.

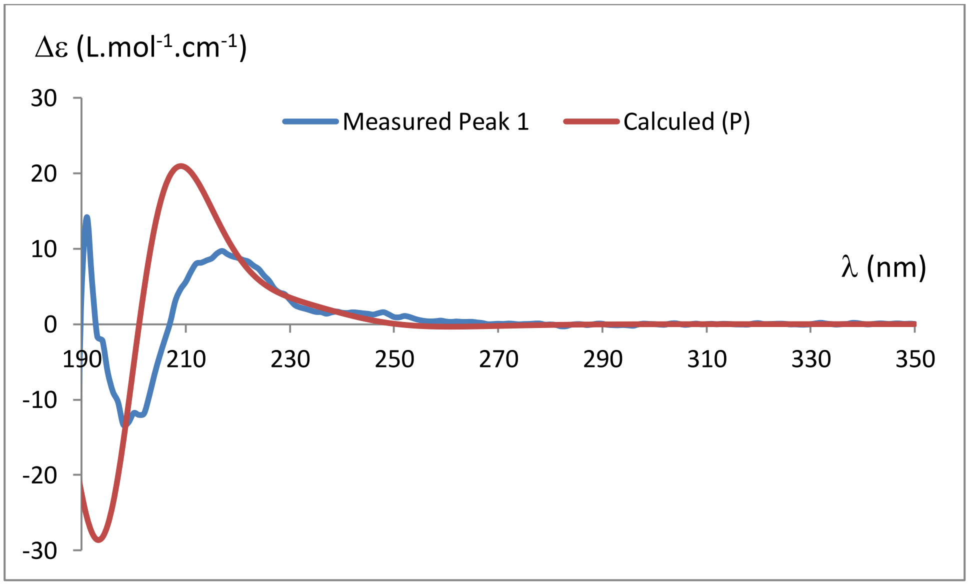

Figure S28. Comparison of measured and calculated ECD spectra for (*P*)-**10**.

S7. Inhibition of WT-TTR in the presence of (*M*)-9 and (*P*)-9 tested at different concentrations

[inhibitor] [μM]	FF%		
	Diflunisal	(*M*)-**9**	(*P*)-**9**
0	100	100	100
0,9	81	97	90
1,8	59	85	88
2,7	33	73	75
3,6	18	49	49
5,4	6	38	47
7,2	6	16	17
10,8	2	15	17
14,5	2	11	7

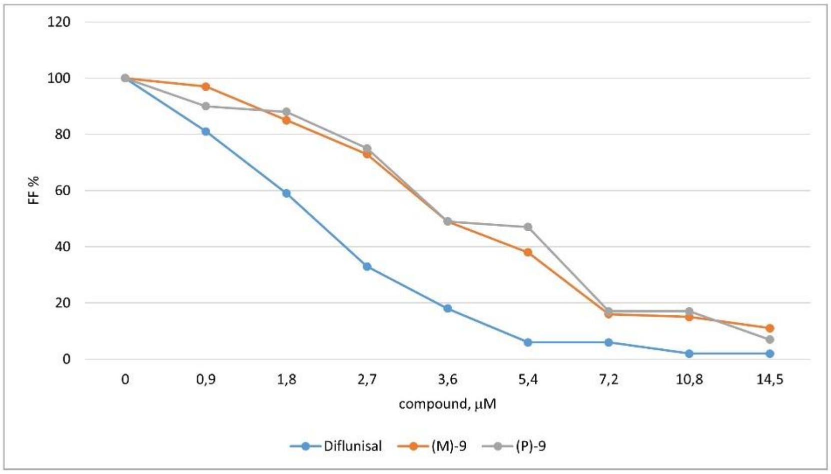

Figure S29. WT-TTR (3.6 μM) was incubated in the presence of different concentrations of (*M*)-**9** (orange line) and (*P*)-**9** (grey line) and the % of fibril formation (FF%) under acidic conditions was measured under standard conditions. Diflunisal (blue line) was also tested as a term of comparison.
